# A Pathogen Penalty? Associations Between Persistent Infections and Biological Aging in the US

**DOI:** 10.1093/infdis/jiaf606

**Published:** 2025-12-11

**Authors:** Jennifer Momkus, Kathleen Mullan Harris, Jessie K Edwards, Yang Claire Yang, Chantel L Martin, Allison E Aiello

**Affiliations:** Department of Epidemiology, Gillings School of Global Public Health, University of North Carolina at Chapel Hill, Chapel Hill, North Carolina, USA; Carolina Population Center, University of North Carolina at Chapel Hill, Chapel Hill, North Carolina, USA; Carolina Population Center, University of North Carolina at Chapel Hill, Chapel Hill, North Carolina, USA; Department of Sociology, University of North Carolina at Chapel Hill, Chapel Hill, North Carolina, USA; Department of Epidemiology, Gillings School of Global Public Health, University of North Carolina at Chapel Hill, Chapel Hill, North Carolina, USA; Carolina Population Center, University of North Carolina at Chapel Hill, Chapel Hill, North Carolina, USA; Carolina Population Center, University of North Carolina at Chapel Hill, Chapel Hill, North Carolina, USA; Department of Sociology, University of North Carolina at Chapel Hill, Chapel Hill, North Carolina, USA; Lineberger Comprehensive Cancer Center, University of North Carolina at Chapel Hill, Chapel Hill, North Carolina, USA; Department of Epidemiology, Gillings School of Global Public Health, University of North Carolina at Chapel Hill, Chapel Hill, North Carolina, USA; Carolina Population Center, University of North Carolina at Chapel Hill, Chapel Hill, North Carolina, USA; Robert N. Butler Columbia Aging Center, Columbia University, New York City, New York, USA; Department of Epidemiology, Mailman School of Public Health, Columbia University, New York City, New York, USA

**Keywords:** persistent infections, cytomegalovirus, epigenetic age acceleration, immunosenescence, aging

## Abstract

**Background:**

Persistent infections, including cytomegalovirus (CMV), herpes simplex virus type 1 (HSV-1), Epstein–Barr Virus (EBV), and *Helicobacter pylori* (*H. pylori*), illicit chronic immune stimulation and may contribute to biological aging. While CMV has been associated with markers of biological aging in older adults, including immunosenescence, less is known about these associations earlier in adulthood or the role of other persistent infections.

**Methods:**

Using data from a nationally representative U.S. cohort, we examined associations between CMV, HSV-1, EBV, and *H. pylori* infections (assessed at a median age of 28 years) and markers of biological aging, including epigenetic age acceleration (EAA) and cellular immunosenescence (measured approximately 10 years later). EAA was assessed via GrimAge, PhenoAge, and DunedinPACE clocks while immunosenescence was estimated using DNA methylation-based immune cell ratios.

**Results:**

CMV infection and antibody concentrations were consistently associated with accelerated epigenetic aging and increased cellular immunosenescence measures. For example, CMV seropositivity was associated with 0.36 higher CD4 + memory: naive ratio (95% CI: .11, .62). *H. pylori*, HSV-1, and EBV demonstrated more limited but notable associations, particularly with EAA measures. For instance, increased EBV IgG was associated with higher GrimAge acceleration (GrimAgeAA) (β=0.006 years, 95% CI: .002, .01). Higher *H. pylori* IgG antibodies were unexpectedly associated with a higher CD4+/CD8 + cell ratio (β=0.002, 95% CI: .0002, .004).

**Conclusions:**

Persistent infections, particularly CMV, shape biological aging via DNA methylation aging and immunosenescence before midlife. Future research is needed to clarify how the timing and burden of these infections influence biological aging and immune function across the life course.

## Teaser

This study examines CMV, HSV-1, EBV, and *H. pylori* infections in U.S. adults before midlife, finding CMV most strongly associated with accelerated epigenetic aging and cellular immunosenescence. Results highlight infection-related contributions to accelerated aging trajectories and potential health disparities.

**(See the Editorial Commentary by Boeckh and Corey on pages e1307–9.)**

Persistent infections, such as cytomegalovirus (CMV), herpes simplex virus-1 (HSV-1), Epstein–Barr virus (EBV), and Helicobacter pylori (*H. pylori*), are widespread and impose chronic immune activation with long-term implications for immune aging and broader biological aging processes [1–3]. CMV, HSV-1, and EBV are herpesviruses usually acquired in youth and establish lifelong latent infections, with periodic reactivations driven by host and environmental factors [1, 4]. *H. pylori* is also often acquired at young ages and colonizes the gastric mucosa, leading to chronic infection [5]. Although eradication is possible with antibiotic therapy, asymptomatic presentation and lack of medical intervention result in persistence for many individuals [5]. Infections with these pathogens are found disproportionately among economically disadvantaged groups, reflecting social patterning in exposure, immune response, and treatment [6].

The impact of persistent infections on incremental systemic aging mechanisms remains poorly understood, especially in younger adult populations. Second- and third-generation epigenetic clocks, which estimate biological age based on DNA methylation patterns, have emerged as robust biomarkers of phenotypic aging before clinical disease onset [7]. While preliminary evidence suggests that CMV may accelerate biological aging [8, 9], limited research has examined these relationships in younger, diverse populations, or explored the roles of HSV-1, EBV, and *H. pylori*. All these infections have been linked to aging-related pathophysiology: *H. pylori* has been linked to chronic inflammation, oxidative stress, and gastric cancers [10], HSV-1 to Alzheimer's Disease and cognitive aging through mechanisms such as neuroinflammation, oxidative stress, and mitochondrial damage [11], and EBV to B-cell lymphomas [12] and neurodegenerative diseases such as multiple sclerosis [13–15]. CMV has been extensively studied in relation to immunosenescence and aging markers such as telomere shortening and earlier epigenetic clocks [8, 16]. It has also been associated with age-related diseases such as cardiovascular diseases, dementia, and cancers [1, 17–19]. These processes may interact with or contribute to broader biological aging mechanisms, potentially accelerating aging trajectories and increasing susceptibility to age-related diseases [20, 21]. A better understanding of how persistent infections influence aging biomarkers early in the life course is also relevant for addressing health disparities, as accelerated biological aging has been hypothesized to mediate socioeconomic inequalities in morbidity and mortality [22].

A hallmark of immunosenescence is the shift in T-cell subsets, characterized by increased memory T cells and depletion of naive T cells [2]. CMV plays a major role in driving memory cell inflation in older adults (≥50 years), particularly CD8 + memory cells, though CD4 + memory cells are also affected [1, 2, 23]. However, less is known about the relationships between CMV and cellular immunosenescence in younger adult U.S. population samples before age 50 [23–25]. Moreover, the impact of other persistent infections on immunosenescence remains underexplored [1, 2]. Studies suggest HSV-1 and EBV may contribute to low-level chronic immune stimulation, but unlike CMV, they are not consistently linked to immunosenescence or age-related diseases [1, 2]. Nevertheless, all require constant immune surveillance and may reactivate when immunity is suppressed [2]. Studies on *H. pylori* have been much more limited and have typically focused on chronic inflammation rather than cellular immunosenescence [26, 27].

In this study, we assessed the associations between CMV, HSV-1. EBV, and *H. pylori* infections, measured at a median age of 28 years, and epigenetic estimates of biological age acceleration and cellular immunosenescence approximately 10 years later in a U.S. nationally representative cohort. By investigating these associations prior to midlife, we aimed to test the hypothesis that persistent infections may contribute to accelerated aging trajectories well before the onset of age-related diseases. Our findings may provide insights into early life contributors to aging and inform strategies for mitigating infection-related health disparities over the life course.

## METHODS

Data are from the National Longitudinal Study of Adolescent to Adult Health (Add Health), a nationally representative U.S. cohort that followed participants from adolescence into adulthood across five waves. Wave IV (2008; median age 28) included interviews, physical exams, and dried blood spot (DBS) collection. Wave V (2016–2018; median age 38) included web, mail, and in-person surveys, along with a separate home exam involving venous blood collection, hereafter “Wave V biosample” (*N* = 5381). Survey weights account for study design, attrition, and participation in the specialized visit. The analytic subsample included participants who provided blood in Wave V (*N* = 4835) and had archived Wave IV DBS (*N* = 3663). Because infection antibody assays were added later, only participants with sufficient archived Wave IV DBS were included (*N* = 971), of whom *N* = 946 had complete covariate data (see Supplementary Section I). Due to small cell sizes, participants identifying as “Pacific Islander,” “Some other race or origin,” or “American Indian/Alaska Native” were excluded (N = 11; see Supplementary Section II), resulting in a final analytic sample of *N* = 935. Further details on the study design and protocols are available in Harris et al [28] and on the Add Health website (https://addhealth.cpc.unc.edu/).

## MEASURES

### Infection Measures

While IgG levels are not a direct marker of reactivation, they can reflect cumulative antigenic stimulation and the degree of immune activity required to maintain latency. Accordingly, higher herpesvirus-specific IgG concentrations are often interpreted as indicators of reduced immune control or greater immune burden [6, 29]. The interpretation of elevated *H. pylori* IgG is less established and cannot distinguish current from past infection. Some research suggests higher *H. pylori* antibody levels are associated with adverse health outcomes [5, 30, 31].

EBV IgG antibodies were measured from Wave IV DBS samples (*N* = 14 687) via enzyme-linked immunosorbent assay (ELISA) (DiaSorin, Stillwater, MN). Based on estimates from NHANES from a similar time period, the top 90% of antibody levels were considered seropositive for EBV (cutoff = 47.2 AU/mL) [29]. A subset of archived DBS samples (*N* = 5019) was later tested for CMV (Diamedix, Miami Lakes, FL), HSV-1 (Focus Diagnostics, Cypress, CA), and *H. pylori* (Abnova, Taipei City, Taiwan) IgG antibodies using ELISA. Assays were validated for use with DBS, with seropositivity thresholds of 46, 1.43, and 13.217 EU/mL, respectively. Detailed protocols are available in the Add Health user guides [32, 33]. Distributions of IgG concentrations are shown in Supplementary Section III.

### Epigenetic Age Acceleration Measures

DNA from Wave V venous blood were assayed at approximately 850 000 CpG sites using the Illumina Infinium MethlyationEPIC BeadChip (Illumina, Inc., San Diego, CA). DNA methylation β values were input into the DNAmAge calculator (https://dnamage.clockfoundation.org/) to estimate second-generation epigenetic clocks: PhenoAge and GrimAge, which are trained on phenotypic aging outcomes. DNAm age was regressed on chronological age to generate the difference between estimated epigenetic age from chronological age, expressed as years of epigenetic age acceleration (EAA; positive values indicate functionally older physiology than expected for their chronological age) [34–36]. The DunedinPACE algorithm was applied to estimate the rate of biological aging, scaled so 1.0 represents the average rate of aging in the Dunedin Study birth cohort (1 biological year of aging per calendar year). Values above 1 indicate a faster pace of aging and below 1 indicate slower aging; a 0.01 increase reflects 1% faster biological aging [37].

### Cellular Immunosenescence Measures

Cell-type proportions were estimated from DNA methylation using the deconvolution method of Salas et al [38]. This yielded ratio of CD4 + memory:naive, CD8 + memory:naive, and CD4+:CD8 + cells. Higher memory:naive ratios indicate a more aged immune cell profile [39]. Higher CD4+:CD8 + ratio reflect a functionally younger immune system [40]. To account for the right-skewed distributions, all values were log-transformed. For participants with estimated cell proportions of 0, a small constant (0.001) was added to all observations to avoid division by zero.

### Covariates

Confounders were selected using a directed acyclic graph and included chronological age, sex assigned at birth (male vs female), immigrant generation (first vs second vs third generation+), neighborhood disadvantage in adolescence, Wave I self-rated health, Wave IV education, and race/ethnicity. Neighborhood disadvantage was derived from 1990 Census tract-level data linked to participants’ 1994–1995 addresses [41]. Wave I self-rated health is used as a proxy of baseline health in early life. Race/ethnicity was self-reported at Wave V. Final categories included “Asian,” “Black/African American,” “Hispanic,” and “White.” Additional details on race/ethnicity are available in Supplementary Section II.

### Analytical Approach

Weighted multivariate linear regression models, adjusted for confounders, estimated associations between Wave IV infections and Wave V cellular immunosenescence and EAA. The parameter of interest was the regression coefficient (β) for the infection exposure, either a binary indicator of seropositivity (vs seronegative/equivocal) or continuous IgG concentration. Models using IgG concentrations were run in the full sample and among seropositive individuals for each infection. Analyses restricted to seropositive participants reflect the relationship between immune system control of the infection and aging outcomes. Among all participants, associations between the concentration of antibodies and outcome measures reflect the total effect of exposure, susceptibility, and immune response.

To address potential bias from differential missingness, stabilized inverse probability of sampling weights (IPSW) were created for our analytic sample based on age, sex assigned at birth, educational attainment, Wave I self-rated health, Wave V census region, and race and ethnicity (mean weight 0.95, range: 0.54–1.82). See Supplementary Section IV for details on missing data and IPSW. These were multiplied by the Wave V biosample survey weights to make results generalizable to the U.S. population of students enrolled in the 1994–1995 school year. Sensitivity analyses of unexpected *H. pylori* associations are in Supplementary Section V. A comparison of Wave IV DBS and Wave V serum-based CMV measures is in Section VI. To assess confounding by coinfections and infection burden, models including all four infections are in Section VII. Analyses were performed in SAS version 9.4, and figures were created in R version 4.3.1. This study was approved by the UNC-Chapel Hill and Columbia University IRBs, and all participants provided written informed consent in accordance with IRB guidelines.

## RESULTS

The weighted summary statistics for the analytic sample (*N* = 935) and Wave V biosample (*N* = 5188) are in Table 1. Our sample was similar to the full biosample. About 36% had a college degree or higher, 3% identified as Asian, 17% as Black/African American, 8% as Hispanic, and 72% as White. The proportion assigned female (55% vs 51%) and mean GrimAgeAA were higher in our sample (0.8 vs 0.2 years).

**Table 1.** Weighted Sample Characteristics of Overall Biosample and Analytic Sample, National Longitudinal Study of Adolescent to Adult Health (Add Health), Wave V (2016–2018)

	Wave V Biosample^a^ (*N* = 5188)	Analytic Sample^b^ (*N* = 935)
Wave IV age (y)^c^	28.3 (0.12)	28.2 (0.15)
Female^d^	3129, 50.8%	596, 54.6%
Race/ethnicity^d^	…	…
Asian	262, 2.6%	47, 2.8%
Black/African American	1035, 17.6%	149, 16.8%
Hispanic	528, 8.5%	91, 8.1%
White	3363, 71.3%	648,72.3%
Wave IV education^d^	…	…
College degree or higher	2042, 36.1%	368, 35.8%
Some college and/or technical training	2076, 43.6%	396, 43.6%
High school degree or lower	859, 20.3%	171, 20.6%
Immigrant generation^d^	…	…
First generation	257, 4.2%	37, 3.2%
Second generation	639, 9.7%	116, 10.0%
Third generation and higher	4244, 86.2%	782, 86.8%
Missing	48	0
Wave I neighborhood disadvantage^c,e^	25.0 (0.92)	25.4 (1.10)
Missing	255	0
Wave 1 self-rated health^c,f^	2.1 (0.02)	2.1 (0.04)
Missing	1	0
Wave IV infection status^d^		
CMV seropositive	547, 43.2%	432, 43.0%
EBV seropositive	4192, 90.2%	853, 90.9%
HSV-1 seropositive	582, 50.8%	463, 51.0%
*H. pylori* seropositive	226, 18.4%	179, 19.7%
IgG antibody concentrations^c^		
CMV IgG	48.3, 2.3	48.2, 2.7
EBV IgG	151.5, 2.2	152.8, 4.3
HSV-1 IgG	4.3, 0.2	4.3, 0.2
*H. pylori* IgG	13.8, 0.9	14.4, 1.1
Missing^g^	4009	13
Epigenetic age acceleration (EAA)^c^		
PhenoAge acceleration (y)	0.1 (0.12)	0.2 (0.23)
GrimAge acceleration (y)	0.2 (0.15)	0.8 (0.25)
DunedinPACE (y of biological aging per calendar y)	0.98 (0.005)	0.97 (0.007)
Missing	729	0
Immune cell ratios (log-transformed)^c^		
CD4 + memory/naive	0.6 (0.03)	0.7 (0.05)
CD8 + memory/naive	0.8 (0.04)	0.8 (0.07)
CD4+/CD8+	−0.7 (0.01)	−0.6 (0.03)
missing	777	0

American Indian/Alaska Native, Pacific Islander, and some other race or origin were removed from both samples due to small cell sizes.

^a^Weighted using Wave V biosample survey weights.

^b^Weighted using the product of Wave V biosample survey weights and inverse probability of sampling weights.

^c^Continuous variable; values shown as weighted mean (SE).

^d^Categorical variable; values shown as *N*, weighted %.

^e^Neighborhood disadvantage was based on 1990 Census data linked to 1994–1995 addresses and reflects a sum of decile scores (range 0–50) across five indicators: female-headed households, poverty, public assistance, low education, and unemployment.

^f^Self-rated health on a scale from 1 to 5, 1 = excellent, 2 = very good, 3 = good, 4 = fair, 5 = poor.

^g^In Wave V biosample, *N* = 4025 missing results for HSV-1 and *N* = 552 missing EBV results. In the analytic sample, *N* = 13 missing for HSV-1 due to insufficient DBS quantity for testing, *N* = 1 missing result for EBV.

**Figure 1.**
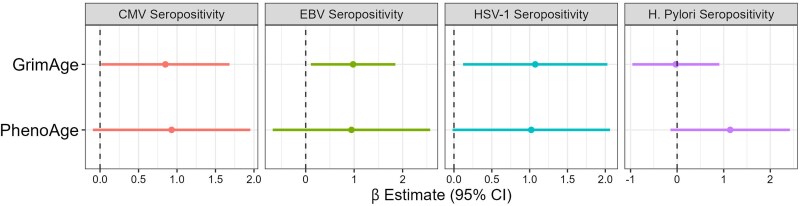
Adjusted associations between persistent infection status and epigenetic age acceleration, Add Health Wave IV-V (*N* = 935). CMV and *H. pylori N* = 935, EBV *N* = 934, HSV-1 *N* = 922. Differences are shown as β estimates and 95% CI's from linear regressions adjusted for age, sex assigned at birth, race/ethnicity, immigrant generation, Wave I neighborhood disadvantage, Wave I self-rated health, and Wave IV educational attainment.

**Figure 2.**
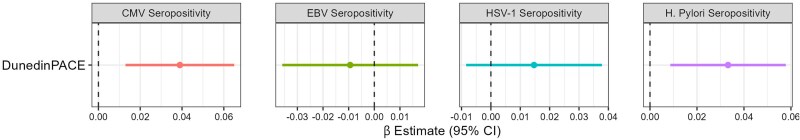
Adjusted associations between persistent infection status and DunedinPACE, Add Health Wave IV-V (*N* = 935). CMV and *H. pylori N* = 935, EBV *N* = 934, and HSV-1 *N* = 922. β estimates and 95% CI's for linear regressions adjusted for age, sex assigned at birth, race/ethnicity, immigrant generation, Wave I neighborhood disadvantage, Wave I self-rated health, and Wave IV educational attainment.

Adjusted associations between infection status and GrimAge acceleration (GrimAgeAA) and PhenoAge acceleration (PhenoAgeAA) are presented in Figure 1; Supplementary Table 19. Associations with DunedinPACE are presented in Figure 2; Supplementary Table 19. CMV seropositivity was associated with higher GrimAgeAA (β=0.85 years, 95% CI: 0.02, 1.68) and DunedinPACE (β = 0.04, 95% CI: .01, .06). EBV and HSV-1 seropositivity were associated with higher GrimAgeAA (EBV: β = 0.98 years, 95% CI: 0.11, 1.85; HSV-1 β=1.07 years, 95% CI: 0.12, 2.03). *H. pylori* was associated with higher DunedinPACE (β=0.03, 95% CI: 0.01, .06).

**Figure 3.**
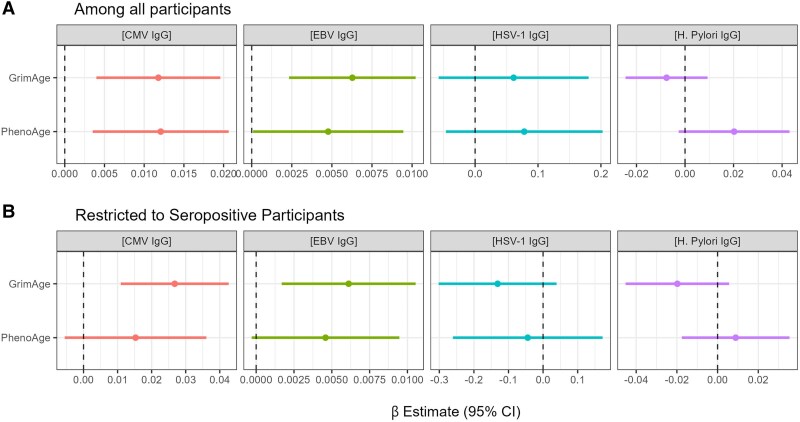
Adjusted associations between infection IgG antibody concentrations and epigenetic age acceleration, Add Health Wave IV-V (N = 935). β estimates and 95% CI's for linear regressions adjusted for age, sex assigned at birth, race/ethnicity, immigrant generation, Wave I neighborhood disadvantage, Wave I self-rated health, and Wave IV educational attainment. *A*) Among all participants: CMV and *H. pylori N* = 935, EBV *N* = 934, and HSV-1 *N* = 922. *B*) Restricted to seropositive participants: CMV *N* = 432, EBV *N* = 853, HSV-1 *N* = 463, and *H. pylori N* = 179.

**Figure 4.**
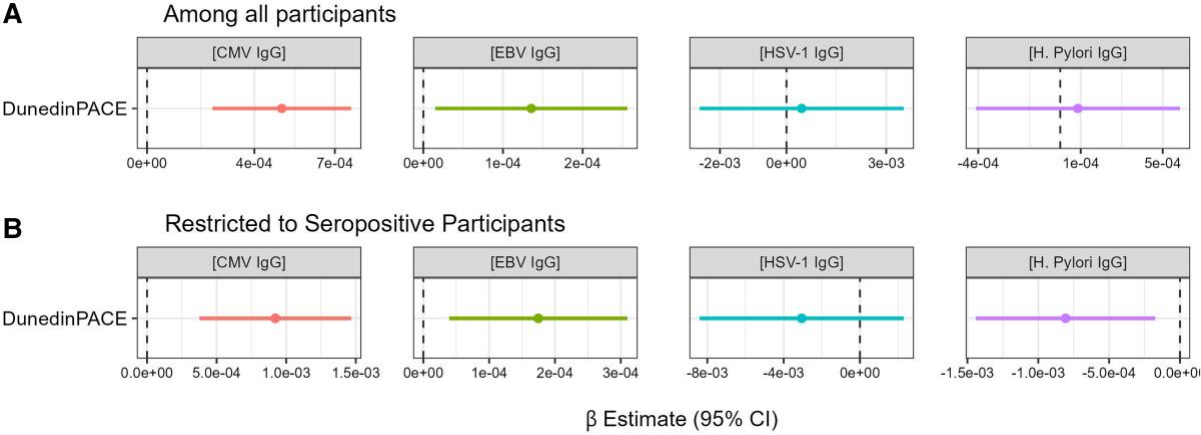
Adjusted associations between infection IgG antibody concentrations and DunedinPACE, Add Health Wave IV-V (*N* = 935). β estimates and 95% CI's for linear regressions adjusted for age, sex assigned at birth, race/ethnicity, immigrant generation, Wave I neighborhood disadvantage, Wave I self-rated health, and Wave IV educational attainment. *A*) Among all participants: CMV and *H. pylori N* = 935, EBV *N* = 934, and HSV-1 *N* = 922. *B*) Restricted to seropositive participants: CMV *N* = 432, EBV *N* = 853, HSV-1 *N* = 463, and *H. pylori N* = 179.

Adjusted associations between IgG concentrations and EAA outcomes are in Figures 3 and 4; Supplementary Table 20. Among all participants, a one-EU/mL increase in CMV IgG was associated with higher GrimAgeAA (β=0.012 years, 95% CI: .0035, .020), PhenoAgeAA (β = 0.012 years, 95% CI: .0035, .021), and DunedinPACE (β=0.0005, 95% CI: .0002, .0008). EBV IgG was associated with all three EAA measures, though associations were generally weaker than CMV (GrimAgeAA: β=0.0063 years, 95% CI: .0023, .010; PhenoAgeAA: β=0.0048 years, 95% CI: .0001, .00945 DunedinPACE: β=0.0001, 95% CI: .000, .0003). *H. pylori* and HSV-1 were not associated with EAA among all participants. Upon restriction to seropositive participants for each infection, CMV and EBV IgG concentrations remained associated with GrimAgeAA (CMV β: 0.027 years, 95% CI: .011, .043; EBV β: 0.0061 years, 95% CI: .0017, .011) and DunedinPACE (CMV β: 0.0009, 95% CI: .0004, .0015; EBV β: 0.0002, 95% CI: .0000, .0003). Among *H. pylori* seropositive participants, higher IgG was associated with lower DunedinPACE (β= −0.0008, 95% CI: −.0014, −.0002).

**Figure 5.**
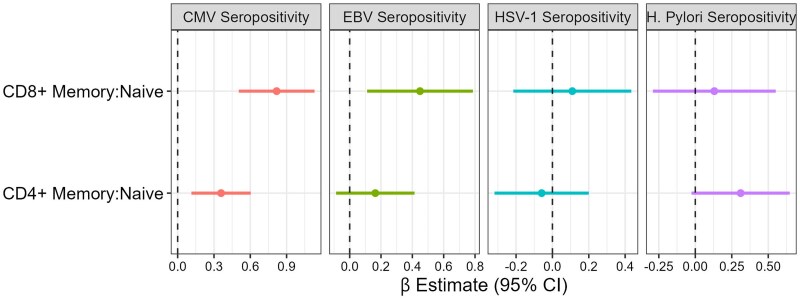
Adjusted associations between persistent infection status and memory/naive cell ratios, Add Health Wave IV-V (*N* = 935). CMV and *H. pylori N* = 935, EBV *N* = 934, HSV-1 *N* = 922. β estimates and 95% CI's for linear regressions adjusted for age, sex assigned at birth, race/ethnicity, immigrant generation, Wave I neighborhood disadvantage, Wave I self-rated health, and Wave IV educational attainment.

Adjusted associations between infection status and T-cell memory: naive ratios are shown in Figure 5; Supplementary Table 19. Associations with CD4+: CD8 + are presented in Supplementary Table 18. CMV infection was associated with higher CD8 + memory/naive (β = 0.82, 95% CI: .50, 1.13) and CD4 + memory: naive ratios (β=0.36, 95% CI: .11, .60), and a lower average CD4+: CD8 + ratio (β= −0.32, 95% CI: −.44, −.21). Associations between other infections and immune cell ratios were less consistent. EBV was associated with a higher CD8 + memory: naive ratio (β=0.45, 95% CI: .11, .79). *H. pylori* seropositivity was linked to a higher CD4 + memory: naive ratio (β=0.31, 95% CI: −.03, .65), though not statistically significant. HSV-1 showed no associations with cellular immunosenescence measures.

**Figure 6.**
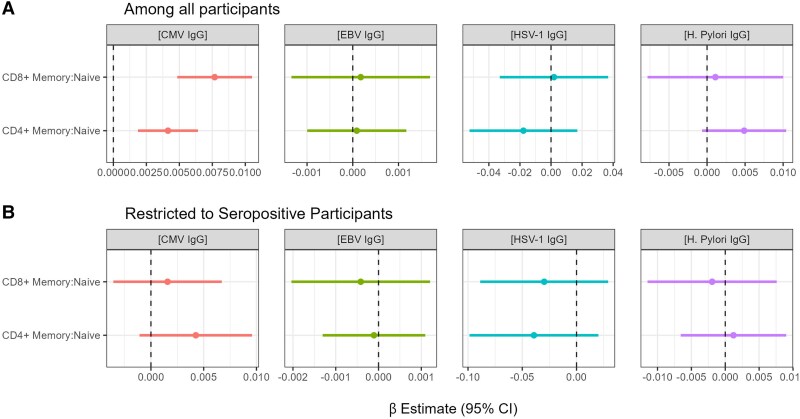
Adjusted associations between infection IgG antibody concentrations and memory/naive cell ratios, Add Health Wave IV-V (*N* = 935). β estimates and 95% CI's for linear regressions adjusted for age, sex assigned at birth, race/ethnicity, immigrant generation, Wave I neighborhood disadvantage, Wave I self-rated health, and Wave IV educational attainment. *A*) Among all participants: CMV and *H. pylori N* = 935, EBV *N* = 934, and HSV-1 *N* = 922. *B*) Restricted to seropositive participants: CMV *N* = 432, EBV *N* = 853, HSV-1 *N* = 463, and *H. pylori N* = 179.

Adjusted associations between antibody concentrations and memory: naive ratios are shown in Figure 6; Supplementary Table 20. Associations with CD4+: CD8 + are in Supplementary Table 18. Among all participants, CMV IgG concentration was associated with all cellular immunosenescence measures (CD8 + memory/naive: β=0.0077, 95% CI: .0048, .011; CD4 + memory/naive: β=0.0041, 95% CI: .0019, .0064; CD4+/CD8+: β=−0.0032, 95% CI: −.0043, −.0022). *H. pylori* IgG was positively associated with the CD4+/CD8 + ratio (full: β= 0.0020, 95% CI: .0002, .0038; seropositive only: β= 0.0028, 95% CI: .0004, .0053). Among seropositive participants, none of the other associations were significant.

## DISCUSSION

This study evaluated the associations between persistent infections—CMV, HSV-1, EBV, and *H. pylori—*and biomarkers of cellular immunosenescence and biological age acceleration in a nationally representative cohort of U.S. adults before midlife. Our findings revealed robust positive associations between CMV seropositivity and IgG antibody concentrations with markers of biological age acceleration and immunosenescence. In contrast, associations for HSV-1, EBV, and *H. pylori* were less consistent and were primarily observed with biological age acceleration rather than immune-specific measures. Notably, this study is among the first to examine these relationships in a diverse younger adult population, before the onset of clinical manifestations of aging. These findings suggest that mechanisms of accelerated aging related to persistent infections, particularly for CMV, may begin well before midlife, highlighting the importance of early life interventions (eg, through vaccination or improved living conditions) to address immune decline and mitigate long-term health disparities.

CMV emerged as the infection most consistently associated with EAA. HSV-1, EBV, and *H. pylori* showed fewer links with T-cell ratios but more frequent associations with EAA, suggesting effects on biological aging independent of T-cell aging. HSV-1 seropositive individuals had, on average, a GrimAgeAA approximately 1 year greater than those who were seronegative or equivocal. A 100 EU/mL increase in EBV antibody concentration (approximately 1 SD) was associated with a 0.5-year increase in PhenoAgeAA, 0.6-year increase in GrimAgeAA, and approximately 1% faster DunedinPACE. For context, these effects are modest compared to the multiyear increases seen with smoking (approximately 6–8 years higher GrimAgeAA, approximately 55–77% increase DunedinPACE) or obesity (approximately 1–3 years higher PhenoAgeAA, approximately 48–68% faster DunedinPACE) [22]. Yet even these modest effect sizes may be meaningful given prior links between small EAA increases and morbidity and mortality [35–37], though EAA remains a biomarker, not a direct predictor of health. *H. pylori* seropositivity was associated with a faster pace of aging as measured by DunedinPACE, but among seropositive individuals, higher *H. pylori* IgG was linked to a slower DunedinPACE. This unexpected finding suggests that the pathophysiological effects of *H. pylori* infection differ from those of herpesviruses and warrants further research. While studies on persistent infections and EAA remain limited, our work corroborates two previous studies linking CMV seropositivity and epigenetic aging in smaller samples of older adults [8, 9]. Further, a study of German adults (50–75 years) found *H. pylori*, particularly CagA + strains, was positively associated with EAA as measured by first-generation clocks [20]. However, these studies did not include the second- and third-generation epigenetic clocks, which are better suited for assessing phenotypic aging [7, 42], nor did they examine antibody concentrations. To our knowledge, no prior studies have assessed the relationships of HSV-1 or EBV with these advanced measures of systemic biological aging.

Previous research has consistently linked herpesvirus infections, particularly CMV, with markers of accelerated cellular immunosenescence in older populations [2, 23, 43, 44]. Our study uniquely incorporates multiple persistent infections, including *H. pylori*, which have been less extensively investigated in relation to T-cell profiles. We observed strong associations between CMV seropositivity and antibody levels with all three immune cell ratios. As expected, associations between CMV and the CD8 + memory/naive ratio were the strongest. Associations between HSV-1, EBV, and *H. pylori* infections with the T-cell ratios were more limited, though there were positive associations between EBV seropositivity and the CD8 + memory: naive ratio. *H. pylori* antibodies were also associated with a higher CD4 + memory: naive ratio, though results were not significant at an alpha level of 0.05. These findings align with three smaller studies in older adults (N < 100, median age > 60) examining multiple herpesviruses and markers of T-cell immunosenescence [45, 46]. Our study extends these findings by leveraging a larger, nationally representative cohort of younger adults, enhancing generalizability to the U.S. population before midlife.

This study provides new insights into the understudied relationship between *H. pylori*, biological age acceleration, and immunosenescence. Unexpectedly, higher *H. pylori* antibody concentrations were linked to a higher CD4+/CD8 + ratio, a marker associated with a functionally younger immune profile. This finding may align with the unexpected association between higher *H. pylori* IgG and a slower pace of aging as measured by DunedinPACE among seropositive individuals. To explore this, we conducted a post hoc sensitivity analysis examining individual immune cell types and observed higher *H. pylori* antibody concentrations were specifically associated with a lower proportion of CD8 + effector memory cells. This may reflect immune modulation by *H. pylori*, such as induction of regulatory T cells that suppress excess gastric inflammation [47]. This immunosuppressive environment may inadvertently inhibit CD8+ T-cell differentiation. Notably, despite the observed relationship with CD8 + memory cells, *H. pylori* antibody concentration was not associated with the CD8 + memory/naive ratio. Nevertheless, these findings may suggest that, once infected, *H. pylori* induces distinct biological and immune processes and merit further investigation.

We noted varying relationships when examining seropositivity versus antibody response among those who were positive for each infection. The contrasting associations observed between serostatus and antibody concentrations with immune-specific and systemic aging outcomes may reflect distinct mechanisms through which persistent infections influence aging processes. CMV seropositivity and antibody concentrations were linked to cellular immunosenescence measures in the full sample but not when restricted to seropositive participants. This may suggest that CMV infection itself, rather than the immune regulation of the infection, may be the critical factor influencing immune aging in this younger cohort. One explanation is that individuals in this age group may not have lived with the infection long enough for substantial expansion of CD8 + and CD4 + memory cell compartments to be associated with antibody responses. Indeed, lifelong priming of the immune system by CMV likely drives the age-related increases in antibody response over time, which has been consistently observed in the literature [2, 48, 49]. In contrast, CMV serostatus and antibody concentrations (both overall and among seropositive individuals) were associated with GrimAgeAA and DunedinPACE, indicating that by early midlife, both the presence of infection and the cumulative immune burden it imposes may play roles in system-wide biological aging.

This study has several limitations. We lack precise data on infection timing and duration, which may influence cumulative immune and biological aging [6, 50]. Further, infection status was measured using IgG antibodies, which do not directly capture pathogen presence or activity. While this provides complementary insight into infection-related aging processes, longitudinal data with repeated measures of infection activity are needed to better characterize these mechanisms. Given the relatively young age and likely intact immune function of our sample, this limitation may be less impactful than in older cohorts. We modeled infections individually, but coinfection may influence susceptibility, immune response, and aging mechanisms. In models including all four infections simultaneously, CMV remained the strongest and most robust predictor, with both seropositivity and higher IgG concentrations significantly associated with all aging outcomes after controlling for the other persistent infections (Supplementary Tables 16-17). Other infections also showed independent effects: HSV-1 infection with higher GrimAgeAA, *H. pylori* infection with higher DunedinPACE, and EBV antibody levels with higher GrimAgeAA and DunedinPACE. Future work should examine possible joint effects of coinfections. Although Add Health is nationally representative, our analytic sample was restricted to those with complete biomarker and confounder data. Survey weights with IPSW were implemented to improve generalizability, but residual selection bias may remain. We conducted sensitivity analyses to assess the impact of missing data and found that associations between CMV and increased cellular immunosenescence are especially robust (Supplementary Figure 5 and 6). Finally, while antibody data were derived from DBS, patterns were consistent with serum-based CMV measures (Supplementary Figure 8-13), reducing concerns about measurement error.

In conclusion, our findings suggest a prominent role of CMV in driving both immune- and system-wide biological aging, while also demonstrating other persistent infections such as HSV-1, EBV, and *H. pylori* may still exert some notable effects. Our ability to observe these relationships earlier in adulthood, prior to the onset of age-related disease, highlights the importance of considering persistent infections as potential contributors to accelerated aging trajectories, especially CMV. Future studies should monitor these relationships longitudinally, assess variation across racial and ethnic groups, consider infection as a mediator of social disparities in health, and explore whether reducing earlier-life infections improves healthspan.

## Supplementary Material

jiaf606_Supplementary_Data

## Supplementary Data

Supplementary materials are available at *The Journal of Infectious Diseases* online (http://jid.oxfordjournals.org/). Supplementary materials consist of data provided by the author that are published to benefit the reader. The posted materials are not copyedited. The contents of all supplementary data are the sole responsibility of the authors. Questions or messages regarding errors should be addressed to the author.
