# Supplementary material for: A Pathogen Penalty? Associations Between Persistent Infections and Biological Aging in the US
Source: J Infect Dis. 2025 Dec 11;233(6):e1320–9. doi: 10.1093/infdis/jiaf606 (PMC13271404; doi:10.1093/infdis/jiaf606)
Supplement: jiaf606_Supplementary_Data [file jiaf606_supplementary_data.docx]

Supplementary Material

[I. Sample selection 2](#_Toc211674781)

[II. Additional Details on Race/Ethnicity 5](#_Toc211674782)

[III. Distributions of Antibody Concentrations 7](#_Toc211674783)

[IV. Assessment of Missing Data and Inverse Probability of Sampling Weights 8](#_Toc211674784)

[V. Post Hoc Investigation of Unexpected Associations with *H. pylori* IgG Concentrations 14](#_Toc211674785)

[VI. Comparison between Wave IV Dried Blood Spot-based CMV measures and Wave V serum-based CMV measure 18](#_Toc211674786)

[VII. Assessment of Infection Burden and Coinfection 22](#_Toc211674787)

[VIII. Associations with CD4+: CD8+ Ratio 26](#_Toc211674788)

[IX. Associations Presented in Figures 1-6 26](#_Toc211674789)

[X. References 30](#_Toc211674790)

# **Sample selection**

Selection into the subsample with blood-based infection data in Wave IV and epigenetic data in Wave V reflected several factors, including participation in both waves (Wave IV response = 80.3%, Wave V = 71.8%), consent, scheduling, and completion of the Wave V biovisit (consent = 65%, response = 67.3%), completion of a blood draw during the biovisit (91.8%), and consent to archive a DBS at Wave IV (75.9%). Further details on design, response, and consent rates are available in Harris 2013 and Harris et al. 2019.^1,2^ From the 11,917 archived DBS samples, 5,019 were assayed for additional markers including persistent infections, based on (1) prioritization of participants in the Wave V biomarker sample and (2) sufficient DBS volume.

Survey weights were created for the Wave V Biosample to account for design, attrition, and participation in this specialized visit. Our analytic sample is a subsample of the weighted Wave V Biosample (see Figure S1 for sample flow chart). The primary source of missing data within the biosample was insufficient archived DBS volume, with other mechanisms described in Section III.

## Figure S1. Sample Flowchart


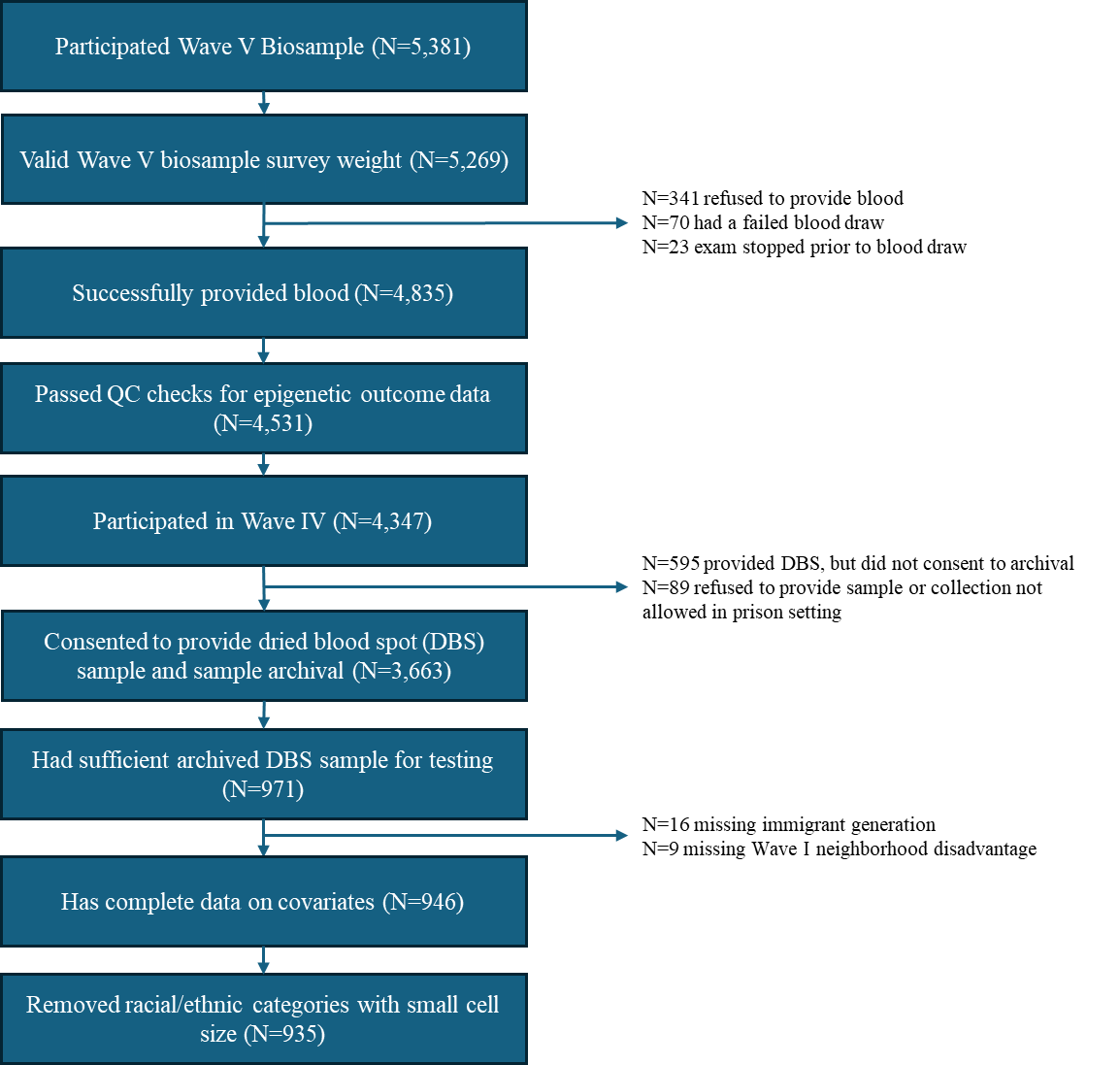


*See Additional Details on Race/Ethnicity

A comparison of the survey-weighted sociodemographic characteristics of those with complete data to those of the overall Wave V biosample is presented in Table S1. The subsample of participants with complete data appears similar to the overall Wave V biosample on all variables except for a higher proportion of female participants (58% vs. 51%) and a higher proportion of white participants (76% vs. 70%). The mean value of GrimAgeAA also appeared slightly higher (0.9 vs. 0.2).

## Table S1. Survey-Weighted Complete Case Sample vs. Overall Cohort Characteristics

|  |  | **Complete Case Sample (N=946)** | | **Wave V Biosample (N=5269)** | |
| --- | --- | --- | --- | --- | --- |
| Wave IV Age | | 28.2 | 15.4% | 28.3 | 0.12 |
| Female | | 606 | 58.3% | 3171 | 50.5% |
| Race/Ethnicity | |  |  |  |  |
|  | American Indian/Alaska Native | -- | -- | -- | -- |
|  | Asian | 47 | 2.7% | 262 | 2.6% |
|  | Black/African American | 149 | 13.3% | 1035 | 17.3% |
|  | Hispanic | 91 | 7.8% | 528 | 8.4% |
|  | Pacific Islander | -- | -- | 27 | 0.2% |
|  | Some other race or origin | -- | -- | 48 | 1.1% |
|  | White | 648 | 75.5% | 3363 | 70.2% |
| Wave IV Education | |  |  |  |  |
|  | College degree or higher | 370 | 34.3% | 2060 | 35.9% |
|  | Some college and/or technical training | 403 | 44.1% | 2113 | 43.5% |
|  | high school degree or lower | 173 | 21.6% | 877 | 20.6% |
| Immigrant Generation | |  |  |  |  |
|  | first generation | 37 | 3.2% | 260 | 4.2% |
|  | second generation | 120 | 10.1% | 659 | 9.8% |
|  | third generation and higher | 789 | 86.7% | 4281 | 86.1% |
|  | missing | 0 |  | 65 |  |
| Wave I Neighborhood Disadvantage | | 25.2 | 1.12 | 25.1 | 0.92 |
|  | missing | 0 |  | 264 |  |
| Wave 1 Self-Rated Health | | 2.1 | 0.05 | 2.1 | 0.02 |
|  | missing | 0 |  | 1 |  |
| Wave IV Infection Status | |  |  |  |  |
|  | CMV Seropositive | 440 | 43.2% | 563 | 43.6% |
|  | HSV-1 Seropositive | 471 | 52.6% | 596 | 51.1% |
|  | *H. pylori* Seropositive | 181 | 19.2% | 230 | 18.4% |
|  | EBV Seropositive | 864 | 91.0% | 4254 | 90.3% |
|  | missing | 13 |  | 4069 |  |
| Immune Cell Ratios (log-transformed) | |  |  |  |  |
|  | CD4 memory/naïve | 0.6 | 0.05 | 0.7 | 0.03 |
|  | CD8 memory/naïve | 0.8 | 0.07 | 0.8 | 0.04 |
|  | CD8/CD4 | -0.7 | 0.03 | -0.7 | 0.01 |
|  | missing | 0 |  | 789 |  |
| Epigenetic Age Acceleration | |  |  |  |  |
|  | PhenoAge | 0.4 | 0.23 | 0.1 | 0.12 |
|  | GrimAge | 0.9 | 0.26 | 0.2 | 0.15 |
|  | DunedinPACE | 1.0 | 0.01 | 1.0 | 0.00 |
|  | missing | 0 |  | 738 |  |

Sample sizes <15 repressed from publication

Statistics shown as N, weighted % for categorical variables and weighted mean, SE for continuous variables.

^1^In the complete case sample: N=13 missing HSV-1 due to insufficient quantity for testing, N=1 missing EBV result. In the overall Wave V biosample, N=4069 missing CMV and *H. pylori*, N=4085 missing HSV-1, and N=569 missing EBV.

# Additional Details on Race/Ethnicity

Race/ethnicity was incorporated as a covariate to serve as a proxy for confounding by systemic impacts of racism, reflecting well-documented associations with disparities in seroprevalence of persistent infections, immune-related dysfunction, and biological aging outcomes.^4–6^ Race/ethnicity was self-reported in Wave V, where respondents selected from predefined categories (“American Indian/Alaska Native,” “Asian,” “Black or African American,” “Hispanic,” “Pacific Islander,” “Some other race or origin,” “White”). Participants selecting multiple categories indicated the group with which they most strongly identified; if Wave V data were missing, responses from Wave I were used.

Several categories had small cell sizes. To evaluate whether groups could be combined, we compared distributions of seroprevalence (Table S2), immune cell ratios, and epigenetic age acceleration (Figure S2) across race/ethnicity. Due to very small numbers and limited comparability, participants identifying as “Pacific Islander,” “Some other race or origin,” or “American Indian/Alaska Native” were excluded (N=11), resulting in a final analytic sample of N=935. These exclusions were made to ensure sufficient statistical power and reliability of inferences.

## Table S2. Seroprevalence of Persistent Infections by Race/Ethnicity

| **Race/Ethnicity** | | **N** | **CMV Seropositive** | **HSV-1 Seropositive** | **EBV Seropositive** | **H. pylori Seropositive** |
| --- | --- | --- | --- | --- | --- | --- |
|  | American Indian/Alaska Native | -- | NA | | | |
|  | Asian | 47 | 62% | 42% | 95% | 19% |
|  | Black/African American | 149 | 67% | 51% | 95% | 33% |
|  | Hispanic | 91 | 60% | 78% | 94% | 26% |
|  | Pacific Islander | -- | 83% | 79% | 100% | 2% |
|  | Some other race or origin | -- | 5% | 97% | 100% | 0% |
|  | White | 648 | 37% | 50% | 90% | 16% |
| Overall | | 946 | 43% | 53% | 91% | 19% |

Sample sizes ≤15 repressed from publication

## Figure S2. Distribution of Outcome Variables by Race/Ethnicity


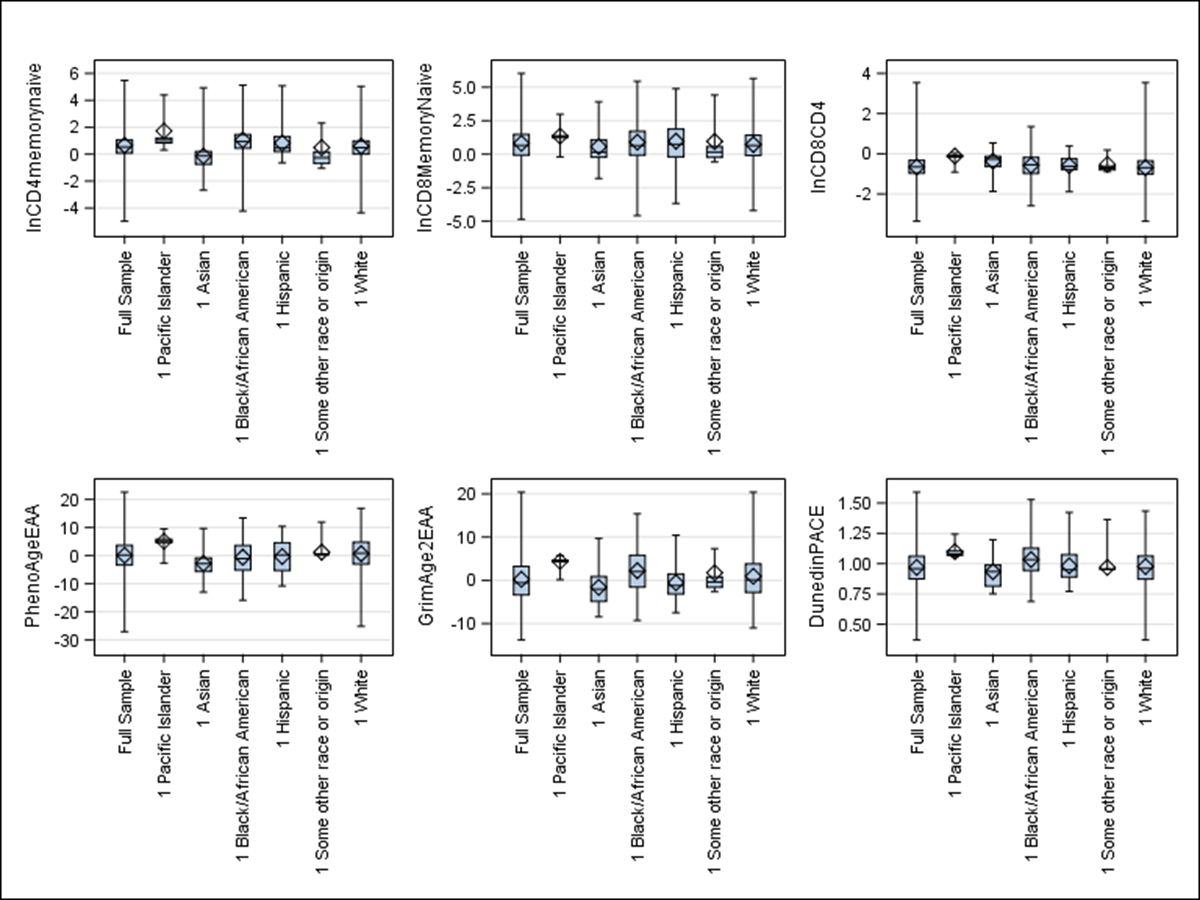


# Distributions of Antibody Concentrations

Below are the distributions of IgG antibody concentrations shown with the unweighted mean and standard deviation for each infection among those who have available data and among those who were included in the final analytic sample (n=935).

Figure S3. Distributions of IgG Antibody Concentrations


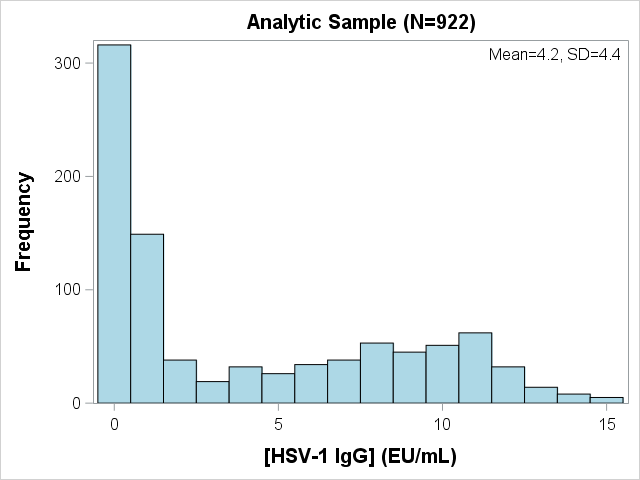

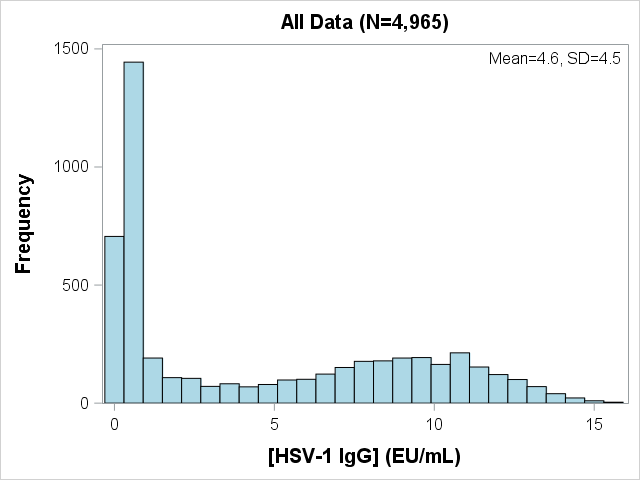

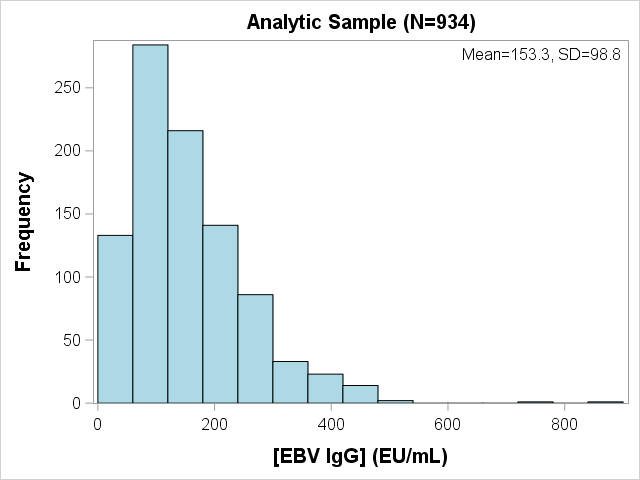

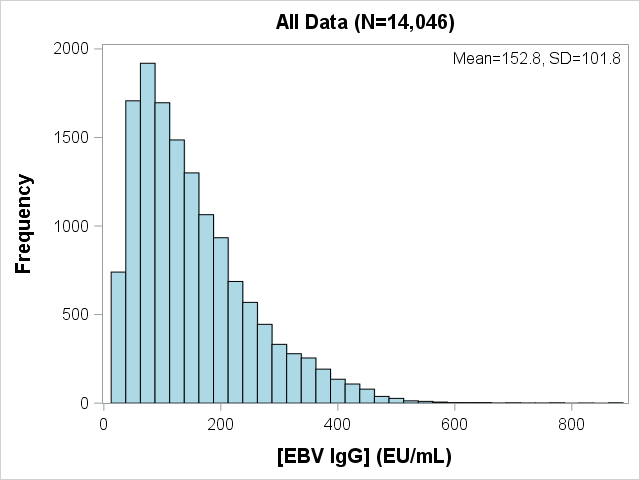

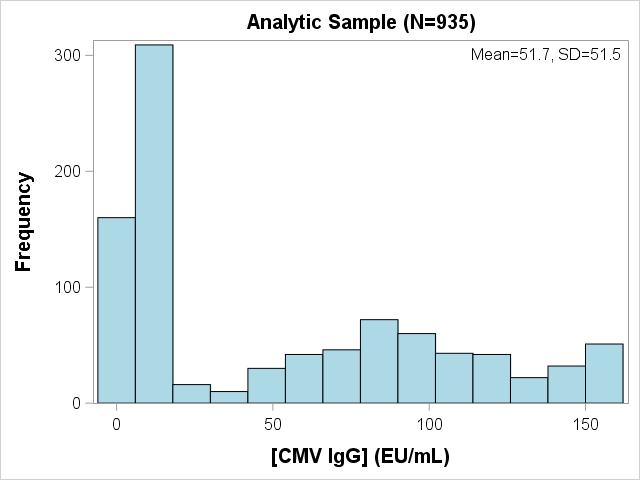

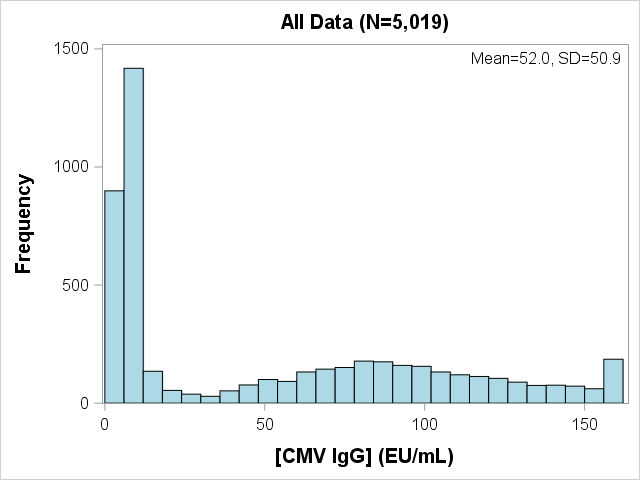

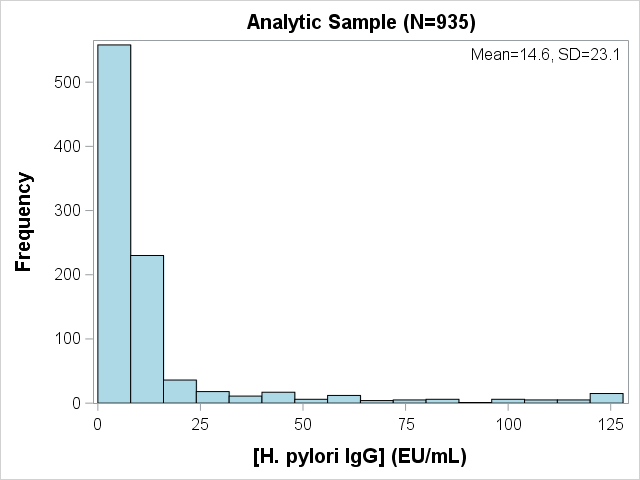

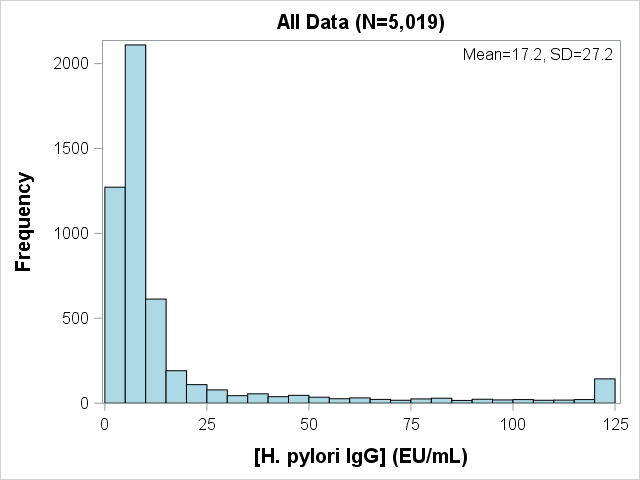


# Assessment of Missing Data and Inverse Probability of Sampling Weights

**Missing Data Mechanisms**

Complete case analysis assumes data are missing completely at random (MCAR). Because most missingness arose from insufficient DBS blood volume, this assumption may be reasonable. However, while infection status itself is unlikely to influence willingness to provide samples, consent may correlate with covariates such as education.^7^ We examined four mechanisms: 1) refusal to provide/archive a Wave IV DBS (Table S3); 2) insufficient archived DBS for testing (Table S4); 3) refusal/inability to provide a Wave V venous sample (Table S5); and 4) quality control exclusions for epigenetic data (Table S6).

Survey-weighted analyses indicated that refusal to provide/archive a Wave IV DBS was not related to outcomes but was associated with age, race/ethnicity, education, and Wave I self-rated health. Insufficient archived DBS was associated with sex and two epigenetic clocks, with males more likely to have insufficient samples and lower GrimAgeAA and DunedinPACE. Missing Wave V blood-based outcomes showed similar patterns: Black/African American participants were less likely to provide venous blood, while younger participants were more likely to fail epigenetic QC. No other study variables were significantly associated with Wave V missingness, though the association with *H. pylori* concentration approached significance (p=0.054). Together, these findings suggest missingness was not fully MCAR, supporting the use of inverse probability of sampling weights (IPSW) to reduce potential bias.

## Table S3. Association between Missing Wave IV Archived Dried Blood Spot and Study Variables, Wave V Biosample N=5050

| **Study Variable** | **Variable Type** | **N** | **p-value*** |
| --- | --- | --- | --- |
| Sex assigned at birth | Categorical | 5050 | 0.37 |
| Race/ethnicity** | Categorical | 4977 | 0.0002 |
| Immigrant generation | Categorical | 4986 | 0.06 |
| Wave IV Educational Attainment | Categorical | 5150 | 0.0007 |
| Age | Continuous | 5150 | 0.04 |
| Wave I Self-Rated Health | Continuous | 5149 | 0.02 |
| Wave I Neighborhood Disadvantage | Continuous | 5005 | 0.40 |
| CD4+ naïve/memory | Continuous | 4347 | 0.53 |
| CD8+ naïve/memory | Continuous | 4347 | 0.19 |
| CD8+/CD4+ | Continuous | 4347 | 0.13 |
| GrimAge | Continuous | 4346 | 0.74 |
| PhenoAge | Continuous | 4346 | 0.48 |
| DunedinPACE | Continuous | 4347 | 0.97 |

*Values are Rao-Scott Chi-Square Test p-values for categorical variables and survey-adjusted t-test p-values for continuous variables

**Restricted to racial/ethnic groups included in analyses

## Table S4. Association Between Insufficient Archived DBS Quantity and Study Variables Among those who consented to archive a Wave IV Dried Blood Spot, Wave V Biosample, N=4186

| **Study Variable** | **Variable Type** | **N** | **p-value*** |
| --- | --- | --- | --- |
| Sex assigned at birth | Categorical | 4186 | 0.003 |
| Race/ethnicity** | Categorical | 4128 | 0.07 |
| Immigrant generation | Categorical | 4134 | 0.43 |
| Wave IV Educational Attainment | Categorical | 4186 | 0.85 |
| Age | Continuous | 4186 | 0.42 |
| Wave I Self-Rated Health | Continuous | 4185 | 0.26 |
| Wave I Neighborhood Disadvantage | Continuous | 4146 | 0.59 |
| CD4+ naïve/memory | Continuous | 3663 | 0.59 |
| CD8+ naïve/memory | Continuous | 3663 | 0.98 |
| CD8+/CD4+ | Continuous | 3663 | 0.77 |
| GrimAge | Continuous | 3662 | 0.001 |
| PhenoAge | Continuous | 3662 | 0.16 |
| DunedinPACE | Continuous | 3663 | 0.01 |

*Values are Rao-Scott Chi-Square Test p-values for categorical variables and survey-adjusted t-test p-values for continuous variables

**Restricted to racial/ethnic groups included in analyses

## Table S5. Association Between Missing Wave V Venous Blood Sample and Study Variables, Wave V Biosample, N=5269

| **Study Variable** | **Variable Type** | **N** | **p-value*** |
| --- | --- | --- | --- |
| Sex assigned at birth | Categorical | 5269 | 0.90 |
| Race/ethnicity** | Categorical | 5188 | 0.04 |
| Immigrant generation | Categorical | 5200 | 0.87 |
| Wave IV Educational Attainment | Categorical | 5050 | 0.83 |
| Age | Continuous | 5050 | 0.75 |
| Wave I Self-Rated Health | Continuous | 5268 | 0.32 |
| Wave I Neighborhood Disadvantage | Continuous | 5005 | 0.58 |
| [CMV IgG] | Continuous | 1200 | 0.64 |
| [HSV-1 IgG] | Continuous | 1184 | 0.38 |
| [*H. pylori* IgG] | Continuous | 1200 | 0.85 |
| [EBV IgG] | Continuous | 4700 | 0.65 |

*Values are Rao-Scott Chi-Square Test p-values for categorical variables and survey-adjusted t-test p-values for continuous variables

**Restricted to racial/ethnic groups included in analyses

## Table S6. Association between Failing Epigenetic Quality Control and Study Variables Among those who provided Wave V Venous Blood, Wave V Biosample (N=4835)

| **Study Variable** | **Variable Type** | **N** | **p-value*** |
| --- | --- | --- | --- |
| Sex assigned at birth | Categorical | 4835 | 0.70 |
| Race/ethnicity** | Categorical | 4761 | 0.29 |
| Immigrant generation | Categorical | 4771 | 0.32 |
| Wave IV Educational Attainment | Categorical | 4640 | 0.49 |
| Age | Continuous | 4640 | 0.009 |
| Wave I Self-Rated Health | Continuous | 4834 | 0.88 |
| Wave I Neighborhood Disadvantage | Continuous | 4596 | 0.61 |
| [CMV IgG] | Continuous | 1064 | 0.92 |
| [HSV-1 IgG] | Continuous | 1048 | 0.38 |
| [*H. pylori* IgG] | Continuous | 1064 | 0.05 |
| [EBV IgG] | Continuous | 4350 | 0.72 |

*Values are Rao-Scott Chi-Square Test p-values for categorical variables and survey-adjusted t-test p-values for continuous variables

**Restricted to racial/ethnic groups included in analyses

**Inverse Probability of Selection Weights**

Because study variables were associated with missingness and the complete case sample differed somewhat from the overall Wave V Biosample, we assumed data were Missing at Random (MAR) and developed stabilized inverse probability of sampling weights (IPSW) for the analytic sample. The IPSW model included age, sex assigned at birth, educational attainment, Wave I self-rated health, Wave V census region, and race/ethnicity. Analyses with and without IPSW produced similar results, though estimates with IPSW were slightly less precise. As expected, the mean stabilized weight was close to 1 (mean = 0.95), with values ranging from 0.54 to 1.82, indicating truncation was not necessary. A histogram of the IPSW and a table comparing sample characteristics before vs. after adding IPSW are shown below.

## Figure S4. Distribution of Inverse Probability of Selection Weights in Analytic Sample


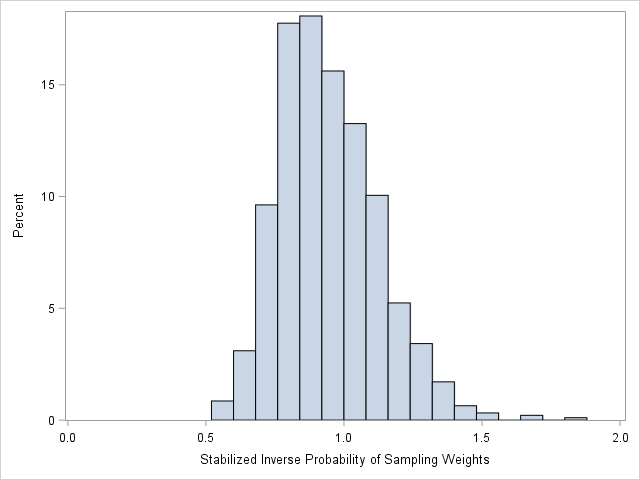


## Table S7. Weighted Sample Characteristics Before and After Adding Inverse Probability of Selection Weights

|  | |  | **No IPSW^1^ (N=935)** | | **With IPSW^2^ (N=935)** | |
| --- | --- | --- | --- | --- | --- | --- |
| Wave IV Age (years) | | | 28.2, | (0.15) | 28.2, | (0.15) |
| Female | | | 596, | 58.2% | 596, | 54.6% |
| Race/Ethnicity | | |  |  |  |  |
|  | Asian | | 47, | 2.7% | 47, | 2.8% |
|  | Black/African American | | 149, | 13.4% | 149, | 16.8% |
|  | Hispanic | | 91, | 7.9% | 91, | 8.1% |
|  | White | | 648, | 76.0% | 648, | 72.3% |
| Wave IV Education | | |  |  |  |  |
|  | College degree or higher | | 368, | 34.5% | 368, | 35.8% |
|  | Some college and/or technical training | | 396, | 44.0% | 396, | 43.6% |
|  | high school degree or lower | | 171, | 21.5% | 171, | 20.6% |
| Immigrant Generation | | |  |  |  |  |
|  | first generation | | 37, | 3.2% | 37, | 3.2% |
|  | second generation | | 116, | 9.9% | 116, | 10.0% |
|  | third generation and higher | | 782, | 86.9% | 782, | 86.8% |
| Wave I Neighborhood Disadvantage^3^ | | | 25.2, | (1.12) | 25.4, | (1.10) |
| Wave 1 Self-Rated Health^4^ | | | 2.1, | (0.05) | 2.1, | (0.04) |
| Wave IV Infection Status | | |  |  |  |  |
|  | CMV Seropositive | | 432, | 43.1% | 432, | 43.0% |
|  | HSV-1 Seropositive | | 463, | 52.4% | 463, | 51.0% |
|  | *H. pylori* Seropositive | | 179, | 19.3% | 179, | 19.7% |
|  | EBV Seropositive | | 853, | 90.9% | 853, | 90.9% |
|  | missing^5^ | | 13 |  | 13 |  |
| Epigenetic Age Acceleration (EAA) | | |  |  |  |  |
|  | PhenoAge Acceleration (years) | | 0.4, | (0.23) | 0.2, | (0.23) |
|  | GrimAge Acceleration (years) | | 0.9, | (0.26) | 0.8, | (0.25) |
|  | DunedinPACE (years of biological aging per calendar year) | | 0.98, | (0.008) | 0.97, | (0.007) |
| Immune Cell Ratios (log-transformed) | | |  |  |  |  |
|  | CD4+ memory/naïve | | 0.6, | (0.05) | 0.7, | (0.05) |
|  | CD8+ memory/naïve | | 0.8, | (0.07) | 0.8, | (0.07) |
|  | CD4+/CD8+ | | -0.7, | (0.03) | -0.6, | (0.03) |

Statistics shown as N, weighted % for categorical variables and weighted mean, SE for continuous variables.

American Indian/Alaska Native, Pacific Islander, and some other race or origin were removed from both samples due to small cell sizes.

^1^Weighted using Wave V Biosample survey weights only

^2^Weighted using the product of Wave V Biosample survey weights and inverse probability of sampling weights

^3^Neighborhood disadvantage was based on 1990 Census data linked to 1994–1995 addresses and reflects a sum of decile scores (range 0–50) across five indicators: female-headed households, poverty, public assistance, low education, and unemployment.

^4^Self-rated health on a scale from 1-5, 1=excellent, 2=very good, 3=good, 4=fair, 5=poor

^5^N=13 missing for HSV-1 due to insufficient DBS quantity for testing, N=1 missing result for EBV.

**Assessment of Missing Data Bias**

To further assess the extent to which missing data may affect results, we also completed sensitivity analyses wherein we assigned values to missing exposure data. We compared the associations where all missing are assigned seropositive vs. a scenario where all missing are assigned seronegative/equivocal. The results from these analyses are below. Though each of the scenarios above is extreme, we see that CMV is still associated with the immune cell ratios, suggesting those results are particularly robust to missing data.

## Figure S5. Associations between Seropositivity and Aging Outcomes, All Missing Infection Data Assigned Seropositive, N=4202


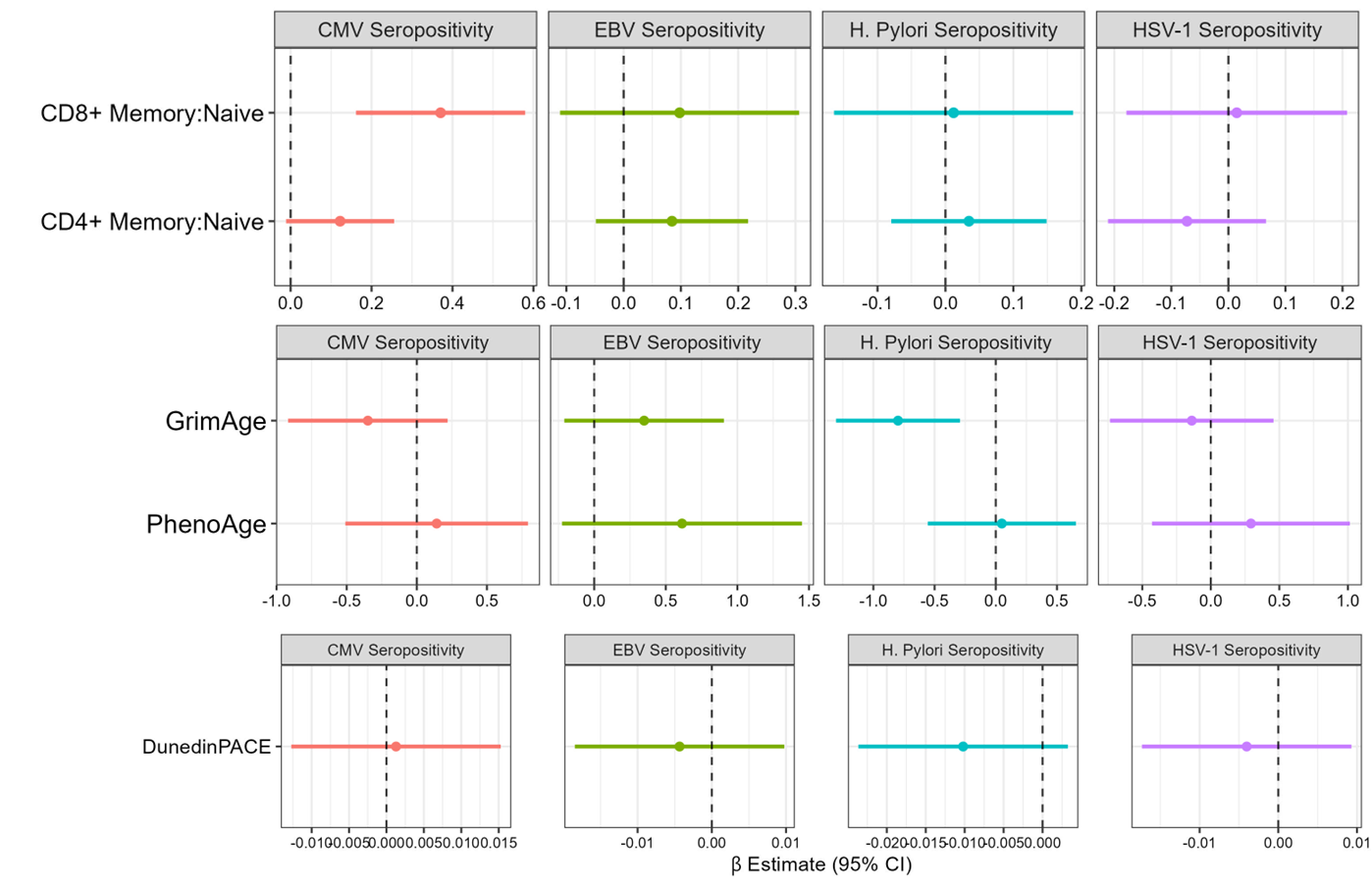
β estimates adjusted for age, sex assigned at birth, race/ethnicity, immigrant generation, Wave I neighborhood disadvantage, Wave I self-rated health, Wave IV education

## Figure S6. Associations between Seropositivity and Aging Outcomes, All Missing Infection Data Assigned Seronegative/Equivocal, N=4202


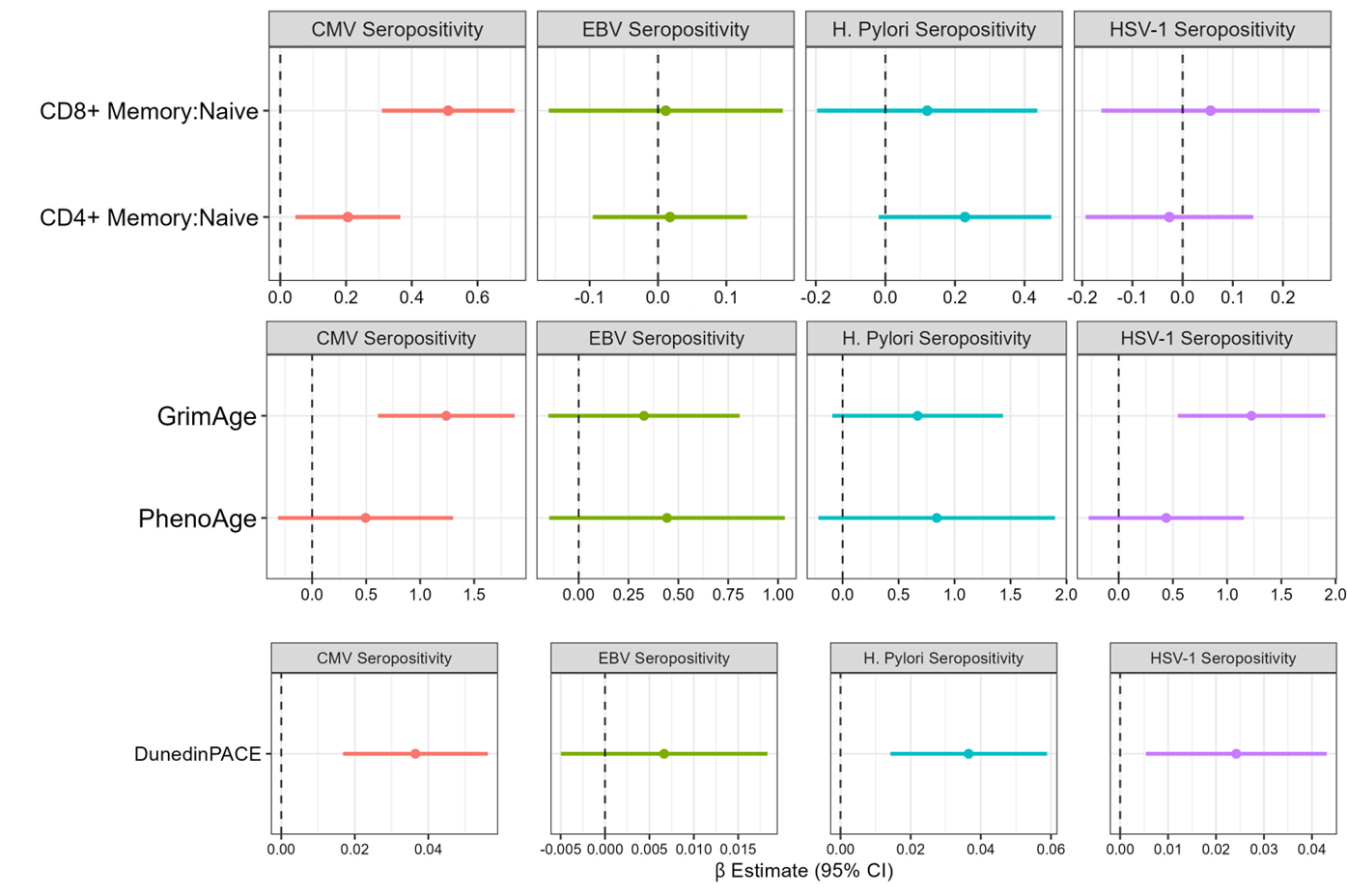
β estimates adjusted for age, sex assigned at birth, race/ethnicity, immigrant generation, Wave I neighborhood disadvantage, Wave I self-rated health, Wave IV education

# Post Hoc Investigation of Unexpected Associations with *H. pylori* IgG Concentrations

We observed a positive association between *H. pylori* antibodies and the CD4+/CD8+ ratio, which contradicted our hypothesis that persistent infections would relate to greater immunosenescence. To explore this further, we examined *H. pylori* antibodies in relation to individual immune cell types. Antibody levels were linked to a lower proportion of CD8+ memory cells in both the full sample and seropositive participants, though not with the CD8+ memory:naïve ratio (Figure S12).

We then tested whether confounding explained these associations by sequentially adjusting for covariates and adding Wave IV BMI (given prior evidence linking *H. pylori* with BMI and BMI with immunosenescence). Associations with lower CD8+ memory proportion persisted across models, strengthening after adjustment for race/ethnicity and immigrant generation (Table S8). In the seropositive sample, all adjusted models were significant despite reduced sample size. Adding BMI did not attenuate effects, suggesting it was not a major confounder (Table S8).

Finally, because *H. pylori* IgG was also associated with slower DunedinPACE among seropositive participants, we repeated the sensitivity analyses. Again, unadjusted models were not significant, but adjustment strengthened the associations, and BMI had no meaningful impact (Table S9).

## Figure S7. Association between *H. pylori* IgG Antibodies and Individual Immune Cell Types, Add Health Wave IV-Wave V, N=935


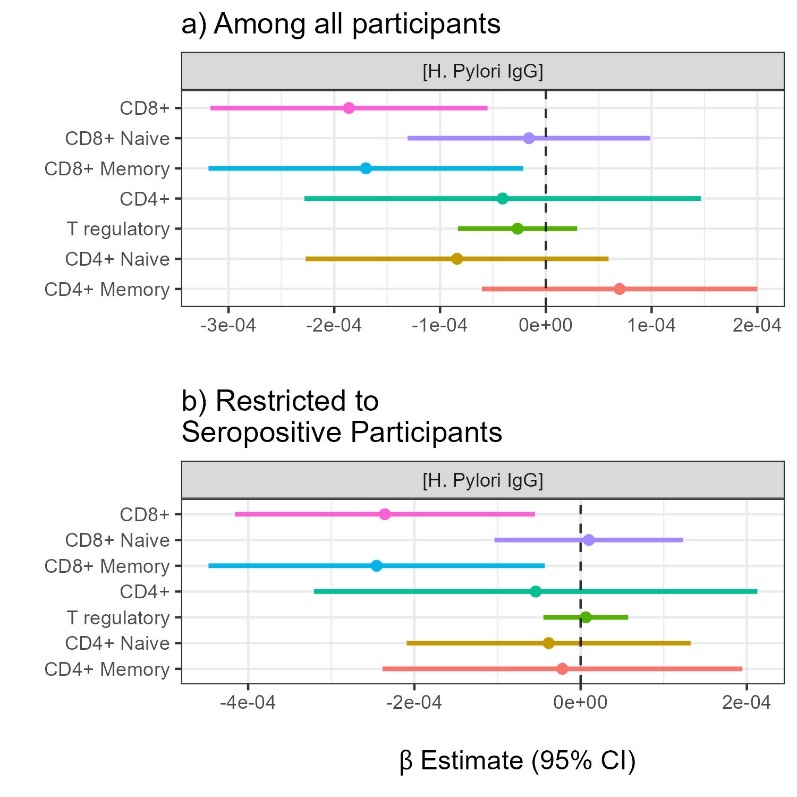


N=935 among all participants, N=179 upon restriction to seropositive participants

Models adjusted for age, sex assigned at birth, race/ethnicity, immigrant generation, Wave I neighborhood disadvantage, Wave I self-rated health, Wave IV education

## Table S8. Association Between [*H. pylori* IgG] and CD8+ Memory Cell Proportion, Add Health Wave IV-Wave V

|  | Among all participants (N=935) | | | Restricted to Seropositive Participants (N=179) | | |
| --- | --- | --- | --- | --- | --- | --- |
| Model Covariates | β | 95% CI | | β | 95% CI | |
| Unadjusted | -6.09E-05 | -2.07E-04 | 8.47E-05 | -1.98E-04 | -4.06E-04 | 9.38E-06 |
| Age | -6.85E-05 | -2.16E-04 | 7.93E-05 | -1.98E-04 | -3.91E-04 | -5.46E-06 |
| Age, sex assigned at birth | -7.83E-05 | -2.33E-04 | 7.65E-05 | -2.16E-04 | -4.22E-04 | -1.00E-05 |
| Age, sex assigned at birth, race/ethnicity | -1.26E-04 | -2.66E-04 | 1.35E-05 | -2.16E-04 | -4.15E-04 | -1.72E-05 |
| Age, sex assigned at birth race/ethnicity, immigrant generation | -1.50E-04 | -2.95E-04 | -4.70E-06 | -2.16E-04 | -4.09E-04 | -2.38E-05 |
| Age, sex assigned at birth race/ethnicity, immigrant generation, Wave I Neighborhood Disadvantage | -1.68E-04 | -3.13E-04 | -2.29E-05 | -2.16E-04 | -4.08E-04 | -2.32E-05 |
| Age, sex assigned at birth race/ethnicity, immigrant generation, Wave I Neighborhood Disadvantage, Wave I self-rated health | -1.71E-04 | -3.18E-04 | -2.47E-05 | -2.45E-04 | -4.44E-04 | -4.60E-05 |
| Age, sex assigned at birth race/ethnicity, immigrant generation, Wave I Neighborhood Disadvantage, Wave I self-rated health, Wave IV education | -1.70E-04 | -3.19E-04 | -2.13E-05 | -2.46E-04 | -4.48E-04 | -4.32E-05 |
| Age, sex assigned at birth race/ethnicity, immigrant generation, Wave I Neighborhood Disadvantage, Wave I self-rated health, Wave IV education, Wave IV BMI | -1.71E-04 | -3.18E-04 | -2.33E-05 | -2.43E-04 | -4.45E-04 | -4.06E-05 |

## Table S9. Association Between [*H. pylori* IgG] and DunedinPACE Restricted to Seropositive Participants, Add Health Wave IV-Wave V, N=179

| Model Covariates | β | 95% CI | |
| --- | --- | --- | --- |
| Unadjusted | -5.91E-04 | -1.27E-03 | 9.04E-05 |
| Age | -7.15E-04 | -1.42E-03 | -6.60E-06 |
| Age, sex assigned at birth | -6.62E-04 | -1.34E-03 | 1.58E-05 |
| Age, sex assigned at birth, race/ethnicity | -7.68E-04 | -1.43E-03 | -1.03E-04 |
| Age, sex assigned at birth race/ethnicity, immigrant generation | -7.75E-04 | -1.43E-03 | -1.18E-04 |
| Age, sex assigned at birth race/ethnicity, immigrant generation, Wave I Neighborhood Disadvantage | -7.75E-04 | -1.44E-03 | -1.07E-04 |
| Age, sex assigned at birth race/ethnicity, immigrant generation, Wave I Neighborhood Disadvantage, Wave I self-rated health | -7.64E-04 | -1.42E-03 | -1.10E-04 |
| Age, sex assigned at birth race/ethnicity, immigrant generation, Wave I Neighborhood Disadvantage, Wave I self-rated health, Wave IV education | -8.07E-04 | -1.44E-03 | -1.75E-04 |
| Age, sex assigned at birth race/ethnicity, immigrant generation, Wave I Neighborhood Disadvantage, Wave I self-rated health, Wave IV education, Wave IV BMI | -8.38E-04 | -1.46E-03 | -2.17E-04 |

# Comparison between Wave IV Dried Blood Spot-based CMV measures and Wave V serum-based CMV measure

Given that dried blood spot-based measures of infection markers have not been as thoroughly validated as serum-based measures, we examined the correlation between Wave IV CMV (measured using dried blood spots) and Wave V CMV (measured using serum samples) and found a strong correlation (N=1041, r=0.78).

We also assessed associations between each CMV measure and Wave V outcomes, finding consistent patterns in significance and effect sizes across measures. This supports the validity of DBS-based CMV assessments as comparable to serum-based measures, though serum data were unavailable for the other infections.

## Figure S8. Comparison of Associations between Wave IV CMV Seropositivity and Epigenetic Age Acceleration with Wave V CMV Seropositivity and Epigenetic Age Acceleration, N=933


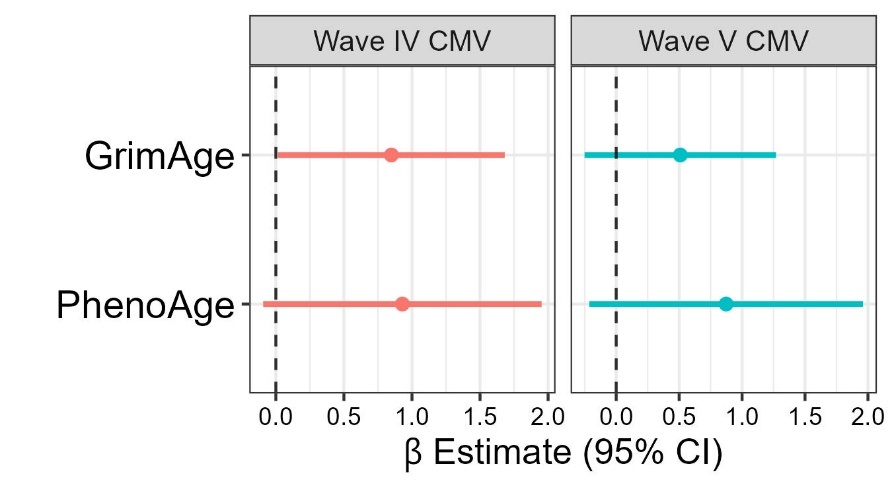


β estimates adjusted for age, sex assigned at birth, race/ethnicity, immigrant generation, Wave I neighborhood disadvantage, Wave I self-rated health, Wave IV education

## Figure 9. Comparison of Associations between Wave IV CMV IgG and Epigenetic Age Acceleration with Wave V CMV IgG and Epigenetic Age Acceleration, N=931


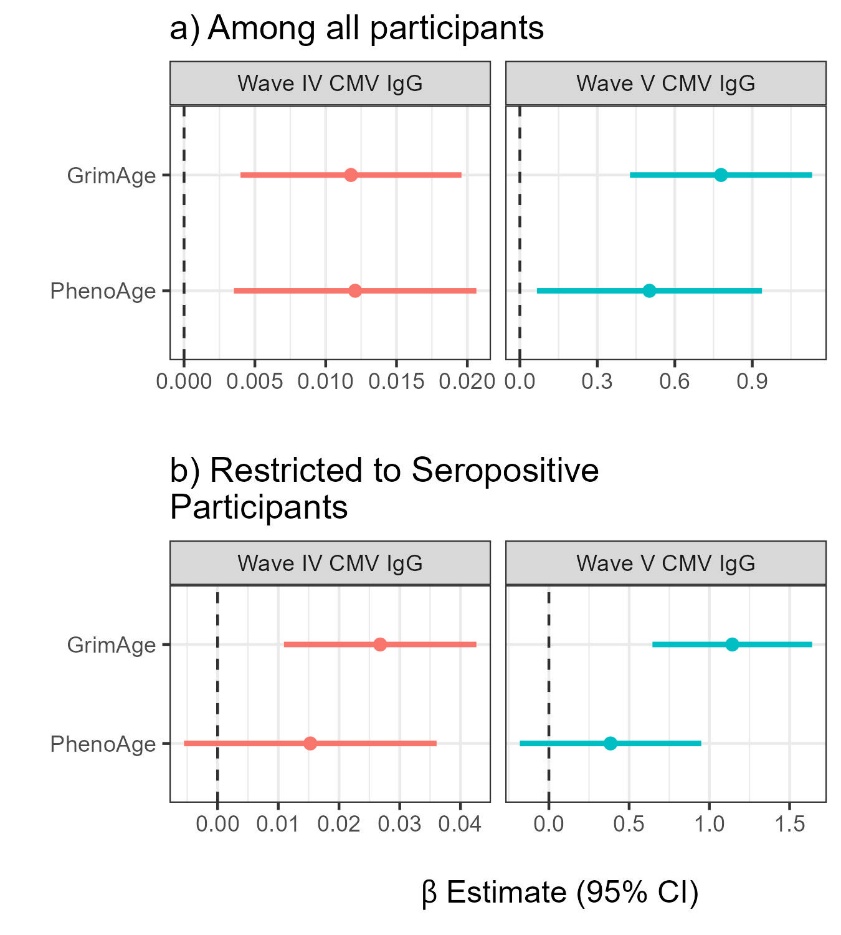


Restricted to seropositive participants in Wave IV N=425, restricted to seropositive participants in Wave V N=505

β estimates adjusted for age, sex assigned at birth, race/ethnicity, immigrant generation, Wave I neighborhood disadvantage, Wave I self-rated health, Wave IV education

## Figure S10. Comparison of Associations between Wave IV CMV Seropositivity and DunedinPACE with Wave V CMV Seropositivity and DunedinPACE, N=933


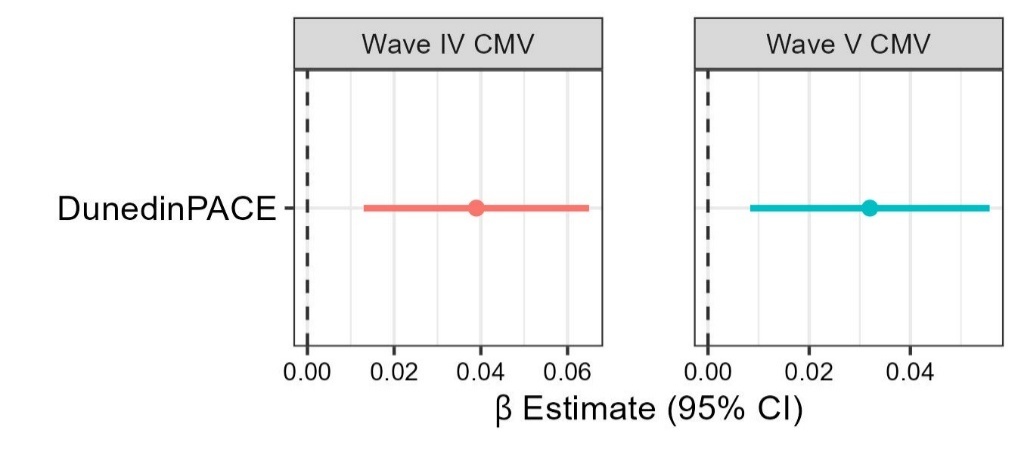


Restricted to seropositive participants in Wave IV N=425, restricted to seropositive participants in Wave V N=505

β estimates adjusted for age, sex assigned at birth, race/ethnicity, immigrant generation, Wave I neighborhood disadvantage, Wave I self-rated health, Wave IV education

## Figure S11. Comparison of Associations between Wave IV CMV IgG and DunedinPACE with Wave V CMV IgG and DunedinPACE, N=931


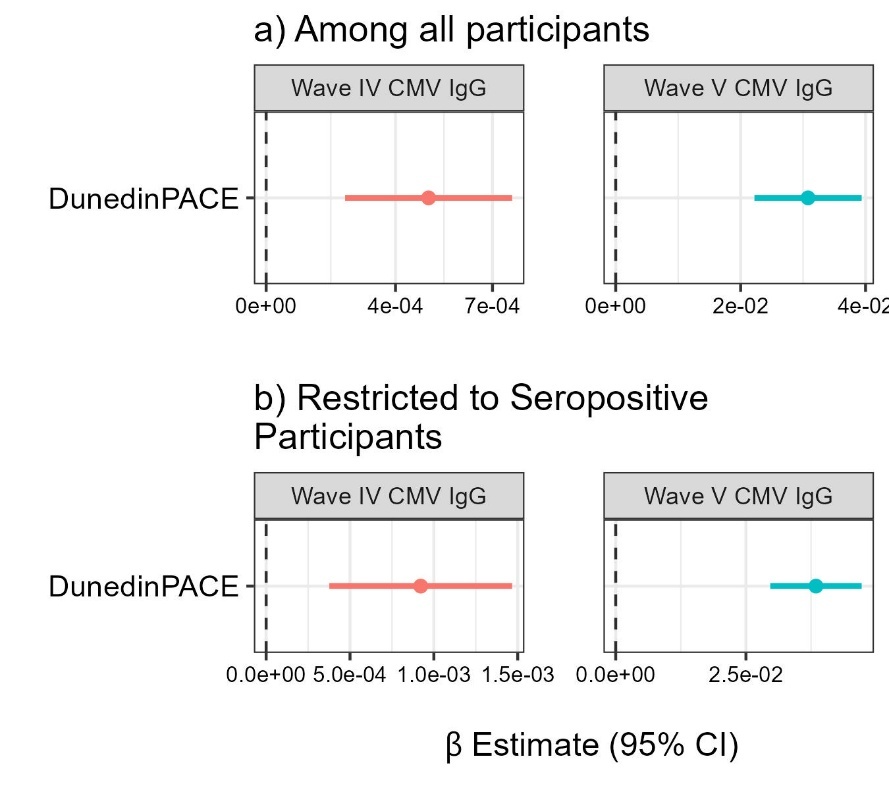


β estimates adjusted for age, sex assigned at birth, race/ethnicity, immigrant generation, Wave I neighborhood disadvantage, Wave I self-rated health, Wave IV education

## Figure S12. Comparison of Associations between Wave IV CMV Seropositivity and Immune Cell Ratios with Wave V CMV Seropositivity and Immune Cell Ratios, N=933


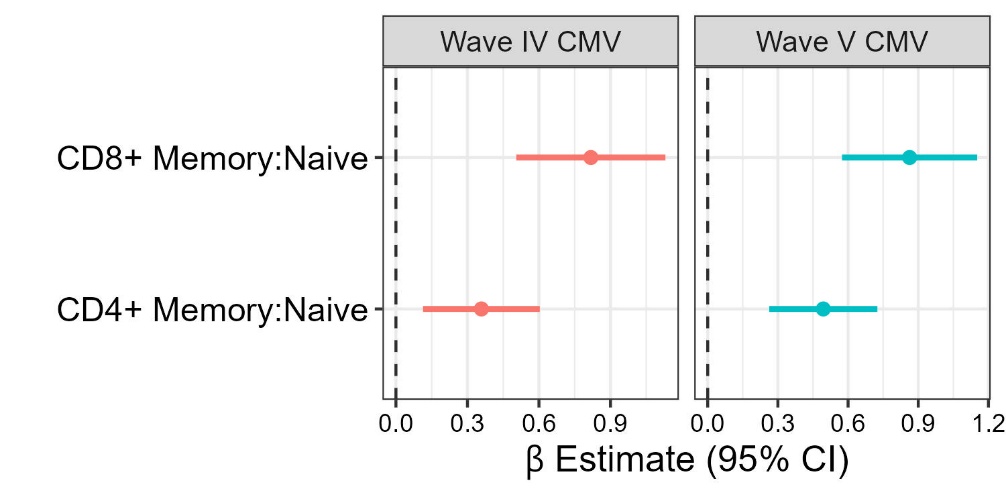


β estimates adjusted for age, sex assigned at birth, race/ethnicity, immigrant generation, Wave I neighborhood disadvantage, Wave I self-rated health, Wave IV education

## Figure S13. Comparison of Associations between Wave IV CMV IgG and Immune Cell Ratios with Wave V CMV IgG and Immune Cell Ratios, N=931


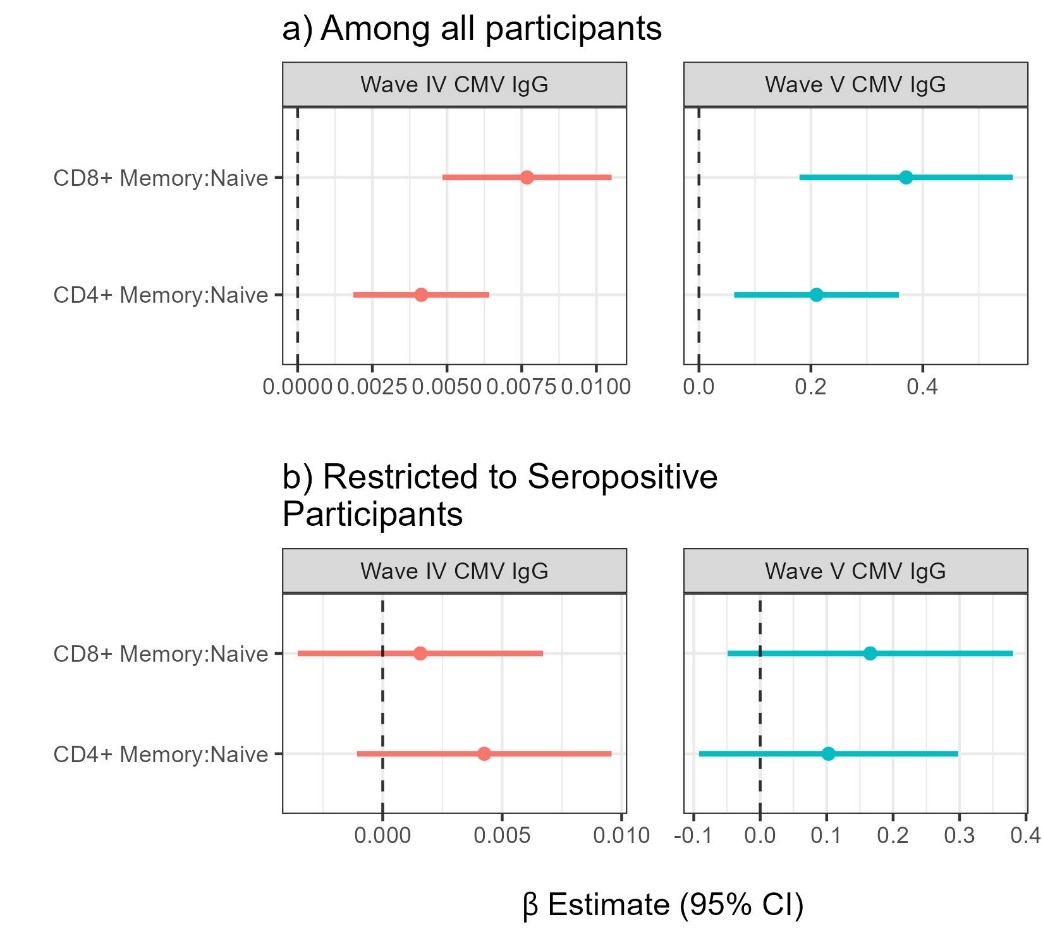


Restricted to seropositive participants in Wave IV N=425, restricted to seropositive participants in Wave V N=505

β estimates adjusted for age, sex assigned at birth, race/ethnicity, immigrant generation, Wave I neighborhood disadvantage, Wave I self-rated health, Wave IV education

# Assessment of Infection Burden and Coinfection

The weighted distribution of infection burden (total number of infections per person) was similar between participants with complete Wave IV infection data and our analytic sample (Table S10). Most participants had at least two infections. Patterns of co-infection were also comparable across groups, with the most common combinations being EBV only, CMV, HSV-1 + EBV, and CMV + EBV (Table S11). To further assess clustering, we examined unadjusted bivariate associations between infections (Tables S12-S15). All infections were significantly associated with each other, except EBV and *H. pylori* (χ² p=0.29, Table S14). Individuals with one infection tended to have higher prevalence of others; for example, HSV-1, EBV, and *H. pylori* were more common among CMV-seropositive participants than among CMV-seronegative.

## Table S10. Distribution of Infection Burden

|  | With available data (N=4670) | | In Analytic Sample (N=921) | |
| --- | --- | --- | --- | --- |
|  | N, | %* | N, | %** |
| Zero Infections | 140, | 3% | 34, | 4% |
| One infection | 1176, | 29% | 236, | 25% |
| Two Infections | 1700, | 36% | 344, | 38% |
| Three Infections | 1264, | 25% | 248, | 27% |
| Four Infections | 390, | 7% | 59, | 5% |

*Wave IV cross-sectional survey-weight applied

**Product of IPSW and Wave V biomarker weight applied in alignment with the rest of analyses

## Table S11. Coinfection Patterns, Add Health Wave IV, N=921

|  | With available data (N=4670) | | In Analytic Sample (N=921) | | |
| --- | --- | --- | --- | --- | --- |
|  | N, | %* | N, | %* |  |
| Zero Infections | 140, | 3% | 34, | 4% |  |
| CMV only | 60, | 1% | 6, | 0.7% |  |
| HSV-1 only | 77, | 2% | 16, | 2% |  |
| EBV only | 1014, | 22% | 211, | 23% |  |
| *H. pylori* Only | 25, | 0.5% | 3, | 0.3% |  |
| CMV + HSV-1 | 55, | 1% | 11, | 1% |  |
| CMV + EBV | 597, | 13% | 132, | 14% |  |
| CMV + *H. pylori* | 16, | 0.3% | 5, | 0.5% |  |
| HSV-1 + EBV | 812, | 17% | 159, | 17% |  |
| HSV-1 + *H. pylori* | 18, | 0.4% | 2, | 0.2% |  |
| EBV + *H. pylori* | 202, | 4% | 35, | 4% |  |
| CMV + HSV-1 + EBV | 847, | 18% | 176, | 19% |  |
| CMV + HSV-1 + *H. pylori* | 32, | 0.7% | 3, | 0.3% |  |
| CMV + EBV + *H. pylori* | 158, | 3% | 33, | 4% |  |
| HSV-1 + EBV + *H. pylori* | 227, | 5% | 36, | 4% |  |
| CMV + HSV-1 + EBV + *H. pylori* | 390, | 8% | 59, | 6% |  |

*Unweighted

## Table S12. Seroprevalence of HSV-1, EBV, and *H. pylori* Stratified by CMV Infection Status

|  | CMV Seropositive (N=2344) | CMV Seronegative/Equivocal (N=2675) | Chi-sq p-value |
| --- | --- | --- | --- |
| HSV-1 Seropositive | 61.8% | 45.7% | <.0001 |
| EBV Seropositive | 92.5% | 89.5% | 0.0002 |
| *H. pylori* Seropositive | 27.8% | 19.0% | <.0001 |

## Table S13. Seroprevalence of CMV, EBV, and *H. pylori* Stratified by HSV-1 Infection Status

|  | HSV-1 Seropositive (N=2641) | HSV-1 Seronegative/Equivocal (N=2324) | Chi-sq p-value |
| --- | --- | --- | --- |
| CMV Seropositive | 54.2% | 38.0% | <.0001 |
| EBV Seropositive | 92.5% | 89.0% | <.0001 |
| *H. pylori* Seropositive | 27.7% | 18.0% | <.0001 |

## Table S14. Seroprevalence of CMV, HSV-1, and *H. pylori* Stratified by EBV Infection Status

|  | EBV Seropositive (N=4557) | EBV Seronegative/Equivocal (N=459) | Chi-sq p-value |
| --- | --- | --- | --- |
| CMV Seropositive | 47.5% | 38.6% | 0.0002 |
| HSV-1 Seropositive | 54.2% | 43.6% | <.0001 |
| *H. pylori* Seropositive | 23.3% | 21.1% | 0.29 |

## Table S15. Seroprevalence of CMV, HSV-1, and EBV Stratified by *H. pylori* Infection Status

|  | *H. pylori* Seropositive (N=1161) | *H. pylori* Seronegative/Equivocal (N=3858) | Chi-sq p-value |
| --- | --- | --- | --- |
| CMV Seropositive | 56.2% | 43.9% | <.0001 |
| HSV-1 Seropositive | 63.6% | 50.1% | <.0001 |
| EBV Seropositive | 91.6% | 90.6% | 0.29 |

Because co-infections may influence susceptibility and immune responses,^57–59^ estimated models including all infections simultaneously, applying the product of Wave V biosample and IPSW weights for generalizability. To assess potential collinearity among infections, we calculated variance inflation factors (VIFs), which were all <2, indicating low multicollinearity. CMV remained the most robust predictor across models, with both seropositivity and higher IgG concentrations significantly associated with all outcomes after controlling for the other persistent infections (see results below). Additionally, HSV-1 infection was independently associated with higher GrimAgeAA, *H. pylori* infection with higher DunedinPACE, and EBV antibody levels with both higher GrimAgeAA and DunedinPACE.

The regression coefficients (β) for each infection were interpreted as the average difference in immune cell ratio and EAA outcomes by infection status conditional on the presence of other infections plus confounders.

## Table S16. Association Between Persistent Infection Status with Cellular Immunosenescence and Epigenetic Age Acceleration Measures when all Infections Included in One Model, N=921

| **Outcome** | **N** | **Infection** | **β Estimate** | **95% CI** | |
| --- | --- | --- | --- | --- | --- |
| ln(CD4+ Memory/Naïve) | 921 | CMV* | 0.37 | 0.12 | 0.63 |
|  | 921 | HSV-1 | -0.10 | -0.36 | 0.17 |
|  | 921 | *H. pylori* | 0.31 | -0.02 | 0.63 |
|  | 921 | EBV | 0.13 | -0.14 | 0.39 |
| ln(CD8+ Memory/Naïve) | 921 | CMV* | 0.83 | 0.50 | 1.16 |
|  | 921 | HSV-1 | 0.01 | -0.33 | 0.35 |
|  | 921 | *H. pylori* | 0.15 | -0.24 | 0.55 |
|  | 921 | EBV | 0.36 | 0.00 | 0.73 |
| ln(CD4+/CD8+) | 921 | CMV* | -0.32 | -0.43 | -0.21 |
|  | 921 | HSV-1 | -0.04 | -0.13 | 0.05 |
|  | 921 | *H. pylori* | 0.05 | -0.06 | 0.16 |
|  | 921 | EBV | -0.08 | -0.32 | 0.15 |
| PhenoAgeAA | 921 | CMV | 0.81 | -0.25 | 1.86 |
|  | 921 | HSV-1 | 0.92 | -0.17 | 2.02 |
|  | 921 | *H. pylori* | 1.06 | -0.22 | 2.34 |
|  | 921 | EBV | 0.68 | -1.07 | 2.43 |
| GrimAgeAA | 921 | CMV | 0.67 | -0.14 | 1.47 |
|  | 921 | HSV-1* | 0.98 | 0.05 | 1.90 |
|  | 921 | *H. pylori* | 0.04 | -0.90 | 0.97 |
|  | 921 | EBV | 0.77 | -0.13 | 1.67 |
| DunedinPACE | 921 | CMV* | 0.04 | 0.01 | 0.06 |
|  | 921 | HSV-1 | 0.01 | -0.01 | 0.03 |
|  | 921 | *H. pylori** | 0.03 | 0.01 | 0.06 |
|  | 921 | EBV | -0.02 | -0.04 | 0.01 |

All models adjusted for age, biological sex, race/ethnicity, immigrant generation, W1 neighborhood disadvantage, Wave I Self-Rated Health, educational attainment in Wave 4. The product of the Wave V biosample weight and inverse probability of sampling weights were applied to all analyses.

*95% CI does not include the null value

To capture both infection and immune control, we repeated analyses using antibody concentrations instead of serostatus. VIFs were all <2, suggesting low collinearity. CMV IgG remained the strongest predictor, significantly associated with immune cell ratios and all epigenetic aging outcomes, independent of other infections. Of note, increased *H. pylori* IgG was associated with a higher CD4+/CD8+ (i.e. reduced cellular immunosenescence) after controlling for antibody levels of other infections, though the lower bound of the 95% CI was 0. In addition, EBV IgG was positively associated with GrimAgeAA and DunedinPACE. HSV-1 was not associated with any of the cellular immunosenescence or EAA outcomes when all infections were included in one model.

## Table S17. Association Between Persistent Infection IgG Concentration with Cellular Immunosenescence and Epigenetic Age Acceleration Measures when all Infections Included in One Model, N=921

| **Outcome** | **N** | **Infection** | **β Estimate** | **95% CI** | |
| --- | --- | --- | --- | --- | --- |
| ln(CD4+ Memory/Naïve) | 921 | [CMV IgG]* | 0.004 | 0.002 | 0.007 |
|  | 921 | [HSV-1 IgG] | -0.021 | -0.055 | 0.014 |
|  | 921 | [*H. pylori* IgG] | 0.005 | -0.001 | 0.011 |
|  | 921 | [EBV IgG] | 0.000 | -0.001 | 0.001 |
| ln(CD8+ Memory/Naïve) | 921 | [CMV IgG]* | 0.008 | 0.005 | 0.011 |
|  | 921 | [HSV-1 IgG] | -0.005 | -0.040 | 0.030 |
|  | 921 | [*H. pylori* IgG] | 0.003 | -0.006 | 0.012 |
|  | 921 | [EBV IgG] | 0.000 | -0.002 | 0.002 |
| ln(CD4+/CD8+) | 921 | [CMV IgG]* | -0.003 | -0.004 | -0.002 |
|  | 921 | [HSV-1 IgG] | -0.002 | -0.011 | 0.007 |
|  | 921 | [*H. pylori* IgG] | 0.002 | 0.000 | 0.003 |
|  | 921 | [EBV IgG] | 0.000 | 0.000 | 0.001 |
| PhenoAgeAA | 921 | [CMV IgG]* | 0.012 | 0.003 | 0.020 |
|  | 921 | [HSV-1 IgG] | 0.066 | -0.058 | 0.190 |
|  | 921 | [*H. pylori* IgG] | 0.019 | -0.003 | 0.040 |
|  | 921 | [EBV IgG] | 0.004 | 0.000 | 0.009 |
| GrimAgeAA | 921 | [CMV IgG]* | 0.011 | 0.003 | 0.018 |
|  | 921 | [HSV-1 IgG] | 0.044 | -0.073 | 0.161 |
|  | 921 | [*H. pylori* IgG] | -0.006 | -0.022 | 0.011 |
|  | 921 | [EBV IgG]* | 0.006 | 0.002 | 0.010 |
| DunedinPACE | 921 | [CMV IgG]* | 5.0E-04 | 2.5E-04 | 7.5E-04 |
|  | 921 | [HSV-1 IgG] | -1.2E-04 | -3.0E-03 | 2.8E-03 |
|  | 921 | [*H. pylori* IgG] | 6.4E-05 | -4.0E-04 | 5.2E-04 |
|  | 921 | [EBV IgG]* | 1.2E-04 | 5.8E-06 | 2.4E-04 |

All models adjusted for age, biological sex, race/ethnicity, immigrant generation, W1 neighborhood disadvantage, Wave I Self-Rated Health, educational attainment in Wave 4. The product of the Wave V biosample weight and inverse probability of sampling weights were applied to all analyses.

*95% CI does not include the null value

# Associations with CD4+: CD8+ Ratio

## Table S18. Adjusted Associations Between Persistent Infections and CD4+: CD8+ Ratio, Add Health Wave IV-Wave V, N=935

| **Sample** | **Sample Size** | **Independent Variable** | **β Estimate** | **95% CI** | |
| --- | --- | --- | --- | --- | --- |
| full | 935 | CMV Seropositivity | -0.32 | (-0.44, | -0.21) |
| full | 935 | [CMV IgG] | -0.0032 | (-0.0043, | -0.0022) |
| Restricted to Seropositive Only | 432 | [CMV IgG] | -0.0008 | (-0.0026, | 0.0010) |
| full | 934 | EBV Seropositivity | -0.12 | (-0.37, | 0.14) |
| full | 934 | [EBV IgG] | 0.0002 | (-0.0004, | 0.0007) |
| Restricted to Seropositive Only | 853 | [EBV IgG] | 0.0004 | (-0.0002, | 0.0009) |
| full | 922 | HSV-1 Seropositivity | -0.07 | (-0.16, | 0.01) |
| full | 922 | [HSV-1 IgG] | -0.0049 | (-0.0137, | 0.0039) |
| Restricted to Seropositive Only | 463 | [HSV-1 IgG] | 0.0072 | (-0.0117, | 0.0261) |
| full | 935 | *H. pylori* Seropositivity | 0.05 | (-0.07, | 0.18) |
| full | 935 | *[H. pylori* IgG*]* | 0.0020 | (0.0002, | 0.0038) |
| Restricted to Seropositive Only | 179 | *[H. pylori* IgG*]* | 0.0028 | (0.0004, | 0.0053) |

CD4+: CD8+ ratios were log-transformed

Associations adjusted for age, sex assigned at birth, immigrant generation, Wave I neighborhood disadvantage, Wave I self-rated health, Wave IV educational attainment, and race/ethnicity

# Associations Presented in Figures 1-6

## Table S19. Associations between Infection Seropositivity with Cellular Immunosenescence and Epigenetic Age Acceleration, Add Health Wave IV-V (N=935)

| **Sample Size** | **Infection** | **Outcome** | **β Estimate*** | **95% CI** | |
| --- | --- | --- | --- | --- | --- |
| 935 | CMV | ln(CD4+Memory/Naïve) | 0.36 | 0.11 | 0.60 |
| 935 | CMV | ln(CD8+ Memory/Naïve) | 0.82 | 0.50 | 1.13 |
| 935 | CMV | PhenoAgeAA | 0.93 | -0.09 | 1.95 |
| 935 | CMV | GrimAgeAA | 0.85 | 0.02 | 1.68 |
| 935 | CMV | DunedinPACE | 0.04 | 0.01 | 0.06 |
| 934 | EBV | ln(CD4+Memory/Naïve) | 0.16 | -0.09 | 0.41 |
| 934 | EBV | ln(CD8+ Memory/Naïve) | 0.45 | 0.11 | 0.79 |
| 934 | EBV | PhenoAgeAA | 0.94 | -0.68 | 2.57 |
| 934 | EBV | GrimAgeAA | 0.98 | 0.11 | 1.85 |
| 934 | EBV | DunedinPACE | -0.01 | -0.04 | 0.02 |
| 935 | *H. pylori* | ln(CD4+Memory/Naïve) | 0.31 | -0.03 | 0.65 |
| 935 | *H. pylori* | ln(CD8+ Memory/Naïve) | 0.13 | -0.29 | 0.55 |
| 935 | *H. pylori* | PhenoAgeAA | 1.14 | -0.14 | 2.42 |
| 935 | *H. pylori* | GrimAgeAA | -0.03 | -0.96 | 0.91 |
| 935 | *H. pylori* | DunedinPACE | 0.03 | 0.01 | 0.06 |
| 922 | HSV-1 | ln(CD4+Memory/Naïve) | -0.06 | -0.32 | 0.20 |
| 922 | HSV-1 | ln(CD8+ Memory/Naïve) | 0.11 | -0.22 | 0.43 |
| 922 | HSV-1 | PhenoAgeAA | 1.02 | -0.02 | 2.06 |
| 922 | HSV-1 | GrimAgeAA | 1.07 | 0.12 | 2.03 |
| 922 | HSV-1 | DunedinPACE | 0.01 | -0.01 | 0.04 |
| *Beta Estimates from survey-weighted linear regressions age, sex, race/ethnicity, immigrant generation, Wave I Neighborhood disadvantage, Wave I self-rated health, and Wave IV education | | | | | |

## Table S20. Associations between IgG Antibodies with Cellular Immunosenescence and Epigenetic Age Acceleration, Add Health Wave IV-V (N=935)

| **Sample Type** | **Sample Size** | **Infection** | **Outcome** | **β Estimate** | **95% CI** | |
| --- | --- | --- | --- | --- | --- | --- |
| full | 935 | CMV | ln(CD4+Memory/Naïve) | 0.0041 | 0.0019 | 0.0064 |
| full | 935 | CMV | ln(CD8+ Memory/Naïve) | 0.0077 | 0.0048 | 0.0105 |
| full | 935 | CMV | PhenoAgeAA | 0.0121 | 0.0035 | 0.0206 |
| full | 935 | CMV | GrimAgeAA | 0.0118 | 0.0040 | 0.0196 |
| full | 935 | CMV | DunedinPACE | 0.0005 | 0.0002 | 0.0008 |
| Seropositive Only | 432 | CMV | ln(CD4+Memory/Naïve) | 0.0043 | -0.0011 | 0.0096 |
| Seropositive Only | 432 | CMV | ln(CD8+ Memory/Naïve) | 0.0016 | -0.0036 | 0.0067 |
| Seropositive Only | 432 | CMV | PhenoAgeAA | 0.0153 | -0.0055 | 0.0361 |
| Seropositive Only | 432 | CMV | GrimAgeAA | 0.0268 | 0.0109 | 0.0426 |
| Seropositive Only | 432 | CMV | DunedinPACE | 0.0009 | 0.0004 | 0.0015 |
| full | 934 | EBV | ln(CD4+Memory/Naïve) | 0.0001 | -0.0010 | 0.0012 |
| full | 934 | EBV | ln(CD8+ Memory/Naïve) | 0.0002 | -0.0013 | 0.0017 |
| full | 934 | EBV | PhenoAgeAA | 0.0048 | 0.0001 | 0.0095 |
| full | 934 | EBV | GrimAgeAA | 0.0063 | 0.0023 | 0.0102 |
| full | 934 | EBV | DunedinPACE | 0.0001 | 0.0000 | 0.0003 |
| Seropositive Only | 853 | EBV | ln(CD4+Memory/Naïve) | -0.0001 | -0.0013 | 0.0011 |
| Seropositive Only | 853 | EBV | ln(CD8+ Memory/Naïve) | -0.0004 | -0.0020 | 0.0012 |
| Seropositive Only | 853 | EBV | PhenoAgeAA | 0.0046 | -0.0003 | 0.0095 |
| Seropositive Only | 853 | EBV | GrimAgeAA | 0.0061 | 0.0017 | 0.0105 |
| Seropositive Only | 853 | EBV | DunedinPACE | 0.0002 | 0.0000 | 0.0003 |
| full | 935 | *H. pylori* | ln(CD4+Memory/Naïve) | 0.0049 | -0.0007 | 0.0104 |
| full | 935 | *H. pylori* | ln(CD8+ Memory/Naïve) | 0.0011 | -0.0078 | 0.0100 |
| full | 935 | *H. pylori* | PhenoAgeAA | 0.0202 | -0.0026 | 0.0431 |
| full | 935 | *H. pylori* | GrimAgeAA | -0.0076 | -0.0245 | 0.0093 |
| full | 935 | *H. pylori* | DunedinPACE | 0.0001 | -0.0004 | 0.0006 |
| Seropositive Only | 179 | *H. pylori* | ln(CD4+Memory/Naïve) | 0.0012 | -0.0066 | 0.0090 |
| Seropositive Only | 179 | *H. pylori* | ln(CD8+ Memory/Naïve) | -0.0019 | -0.0115 | 0.0076 |
| Seropositive Only | 179 | *H. pylori* | PhenoAgeAA | 0.0089 | -0.0176 | 0.0354 |
| Seropositive Only | 179 | *H. pylori* | GrimAgeAA | -0.0198 | -0.0452 | 0.0057 |
| Seropositive Only | 179 | *H. pylori* | DunedinPACE | -0.0008 | -0.0014 | -0.0002 |
| full | 922 | HSV-1 | ln(CD4+Memory/Naïve) | -0.0178 | -0.0525 | 0.0169 |
| full | 922 | HSV-1 | ln(CD8+ Memory/Naïve) | 0.0019 | -0.0330 | 0.0368 |
| full | 922 | HSV-1 | PhenoAgeAA | 0.0781 | -0.0464 | 0.2027 |
| full | 922 | HSV-1 | GrimAgeAA | 0.0613 | -0.0580 | 0.1805 |
| full | 922 | HSV-1 | DunedinPACE | 0.0005 | -0.0026 | 0.0035 |
| Seropositive Only | 463 | HSV-1 | ln(CD4+Memory/Naïve) | -0.0392 | -0.0986 | 0.0202 |
| Seropositive Only | 463 | HSV-1 | ln(CD8+ Memory/Naïve) | -0.0298 | -0.0887 | 0.0291 |
| Seropositive Only | 463 | HSV-1 | PhenoAgeAA | -0.0444 | -0.2608 | 0.1720 |
| Seropositive Only | 463 | HSV-1 | GrimAgeAA | -0.1315 | -0.3019 | 0.0390 |
| Seropositive Only | 463 | HSV-1 | DunedinPACE | -0.0031 | -0.0084 | 0.0023 |

*Beta Estimates from survey-weighted linear regressions age, sex, race/ethnicity, immigrant generation, Wave I Neighborhood disadvantage, and Wave IV education

# References

1. Harris KM, Halpern CT, Whitsel EA, et al. Cohort Profile: The National Longitudinal Study of Adolescent to Adult Health (Add Health). International Journal of Epidemiology 2019; 48: 1415–1415k.

2. Harris KM. The add health study: Design and accomplishments. Chapel Hill: Carolina Population Center, University of North Carolina at Chapel Hill 2013; 1: 1–22.

3. Chen P, Harris KM. Construction of Wave V Biomarker Weight.

4. Dowd JB, Palermo T, Chyu L, et al. Race/ethnic and socioeconomic differences in stress and immune function in The National Longitudinal Study of Adolescent Health. Social Science & Medicine 2014; 115: 49–55.

5. Stebbins RC, Noppert GA, Aiello AE, et al. Persistent socioeconomic and racial and ethnic disparities in pathogen burden in the United States, 1999–2014. Epidemiology & Infection 2019; 147: e301.

6. Noppert GA, Stebbins RC, Dowd JB, et al. Socioeconomic and race/ethnic differences in immunosenescence: Evidence from the Health and Retirement Study. Brain, Behavior, and Immunity 2023; 107: 361–368.

7. Sanderson SC, Brothers KB, Mercaldo ND, et al. Public Attitudes toward Consent and Data Sharing in Biobank Research: A Large Multi-site Experimental Survey in the US. The American Journal of Human Genetics 2017; 100: 414–427.

8. Belsky DW, Domingue BW, Wedow R, et al. Genetic analysis of social-class mobility in five longitudinal studies. Proceedings of the National Academy of Sciences 2018; 115: E7275–E7284.

9. Chen L-W, Kuo S-F, Chen C-H, et al. A community-based study on the association between Helicobacter pylori Infection and obesity. Scientific Reports 2018; 8: 10746.

10. Baradaran A, Dehghanbanadaki H, Naderpour S, et al. The association between Helicobacter pylori and obesity: a systematic review and meta-analysis of case–control studies. Clinical Diabetes and Endocrinology 2021; 7: 15.

11. Shimi G, Sohouli MH, Ghorbani A, et al. The interplay between obesity, immunosenescence, and insulin resistance. Immunity & Ageing 2024; 21: 13.

12. Zajacova A, Dowd JB, Aiello AE. Socioeconomic and Race/Ethnic Patterns in Persistent Infection Burden Among U.S. Adults. The Journals of Gerontology: Series A 2009; 64A: 272–279.

13. Delaney AS, Thomas W, Balfour HH Jr. Coprevalence of Epstein-Barr Virus, Cytomegalovirus, and Herpes Simplex Virus Type-1 Antibodies Among United States Children and Factors Associated With Their Acquisition. Journal of the Pediatric Infectious Diseases Society 2015; 4: 323–329.

14. Indari O, Ghosh S, Bal AS, et al. Awakening the sleeping giant: Epstein–Barr virus reactivation by biological agents. Pathogens and Disease 2024; 82: ftae002.
